# LncRNA HCP5-Encoded Protein Regulates Ferroptosis to Promote the Progression of Triple-Negative Breast Cancer

**DOI:** 10.3390/cancers15061880

**Published:** 2023-03-21

**Authors:** Xiao Tong, Zhengling Yu, Jiani Xing, Haizhou Liu, Shunheng Zhou, Yu’e Huang, Jing Lin, Wei Jiang, Lihong Wang

**Affiliations:** 1 Department of Pathophysiology, Medical College, Southeast University, Nanjing 210009, China; 2 Department of Biomedical Engineering, Nanjing University of Aeronautics and Astronautics, Nanjing 211106, China; 3 Institute of Cancer Prevention and Treatment, Heilongjiang Academy of Medical Science, Harbin Medical University, Harbin 150081, China; 4 Jiangsu Provincial Key Laboratory of Critical Care Medicine, Nanjing 210009, China

**Keywords:** lncRNA HCP5, encoded protein, ferroptosis, ROS, triple-negative breast cancer

## Abstract

**Simple Summary:**

The objective of the study was to investigate the possibility of lncRNA HCP5-encoded peptide/protein and the role of the resulting product in triple-negative breast cancer. Our study revealed that lncRNA HCP5 encodes a 132-amino acid sequence, referred to as HCP5-132aa, which enhances the growth of triple-negative breast cancer cells by regulating ferroptosis. Moreover, breast cancer patients with elevated levels of HCP5-132aa had a worse prognosis. Our findings have revealed a regulatory mechanism of ferroptosis in triple-negative breast cancer that is modulated by an lncRNA-encoded product.

**Abstract:**

Background: Long non-coding RNAs (lncRNAs) are a class of RNA molecules that are longer than 200 nucleotides and were initially believed to lack encoding capability. However, recent research has found open reading frames (ORFs) within lncRNAs, suggesting that they may have coding capacity. Despite this discovery, the mechanisms by which lncRNA-encoded products are involved in cancer are not well understood. The current study aims to investigate whether lncRNA HCP5-encoded products promote triple-negative breast cancer (TNBC) by regulating ferroptosis. Methods: We used bioinformatics to predict the coding capacity of lncRNA HCP5 and conducted molecular biology experiments and a xenograft assay in nude mice to investigate the mechanism of its encoded products. We also evaluated the expression of the HCP5-encoded products in a breast cancer tissue microarray. Results: Our analysis revealed that the ORF in lncRNA HCP5 can encode a protein with 132-amino acid (aa), which we named HCP5-132aa. Further experiments showed that HCP5-132aa promotes TNBC growth by regulating GPX4 expression and lipid ROS level through the ferroptosis pathway. Additionally, we found that the breast cancer patients with high levels of HCP5-132aa have poorer prognosis. Conclusions: Our study suggests that overexpression of lncRNA HCP5-encoded protein is a critical oncogenic event in TNBC, as it regulates ferroptosis. These findings could provide new therapeutic targets for the treatment of TNBC.

## 1. Introduction

A recent report revealed that the most common type of cancers worldwide was female breast cancer with about 2.26 million cases [1]. In China, breast cancer also has the highest incidence among female malignant tumors. Triple-negative breast cancer (TNBC), accounting for 10–17% of cases, is known for its high aggressiveness and poor prognosis due to the lack of effective therapeutic targets [2]. Despite extensive research, the molecular mechanisms underlying TNBC tumorigenesis remain incompletely understood.

Ferroptosis is a type of programmed cell death characterized by the shrinkage of mitochondria, increased membrane density, and decrease or missing mitochondrial cristae in cell morphology [3]. This process is marked by lipid peroxide accumulation, membrane repair, and iron-dependent reactive oxygen species (ROS) accumulation [4,5]. Several genes and pathways have been implicated in the regulation of iron and ROS metabolism during ferroptosis, including the Xc-/GSH/GPX4, ACSL4/LPCAT3/15-LOX and AMID/CoQ10 pathways [6,7,8].

Long non-coding RNAs (lncRNAs) are abundant in various organs and systems of the body [9,10,11]. Despite being considered as genomic “noise” in the past, the advancements in bioinformatics have revealed that 2% of lncRNAs encode proteins or peptides [12]. Studies have shown that lncRNA-encoded proteins or peptides related to tumors can be combined with conventional anticancer drugs or chemoradiotherapy to enhance the therapeutic effect and reduce mortality rates [13]. For example, the ASRPS peptide encoded by lncRNA plays a role in the malignant progression of TNBC, whereas the HOXB-AS3 peptide inhibits colorectal cancer (CRC) growth [14,15]. Moreover, a 73 aa peptide encoded by CircPPP1R12A has been found to facilitate colon cancer metastasis [16]. Recently, an 85aa peptide MBOP encoded by LINC01234 was reported to promote CRC via the MAPK signaling pathway [17].

In this study, we discovered that the lncRNA HLA complex P5 (HCP5), which was previously known as an oncogene in TNBC [18], encodes a 132-amino acid small protein named HCP5-132aa. Overexpression of HCP5-132aa ORF promoted TNBC cell growth and colony formation. On the other hand, knockdown of HCP5-132aa ORF led to reduced HCP5-132aa expression, increased lipid ROS, and induced ferroptosis by decreasing GPX4 in TNBC cells. High levels of the HCP5-132aa protein were associated with a poor survival rate in breast cancer patients. Overall, our findings reveal that an lncRNA-encoded protein, HCP5-132aa, promotes the malignant progression of TNBC by inhibiting ferroptosis.

## 2. Materials and Methods

### 2.1. Prediction of LncRNA HCP5-Encoded Products Using Bioinformatics Analysis

**Figure 1:**
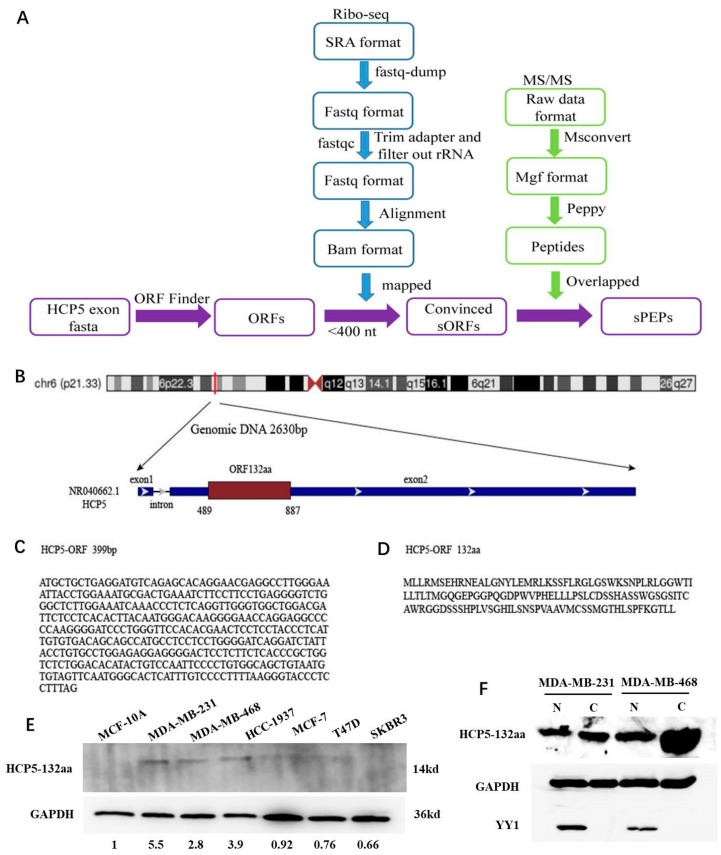
LncRNA HCP5 encoded a protein and endogenously upregulated in TNBC cell lines. (**A**) Workflow diagram of HCP5-encoded sPEPs identification. (**B**) The sketch map of lncRNA HCP5 location. (**C**) The ORF sequence of HCP5. (**D**) The 132-amino acid translated by HCP5-ORF. (**E**) Western blot demonstrating that HCP5-132aa was endogenously expressed in human breast cancer cell lines but higher in TNBC cell lines (MDA-MB-231, MDA-MB-468, and HCC-1937). Single representative western blot is shown from three separate experiments. (**F**) Subcellular localization showed that HCP5-132aa expressed both in the nucleus and cytoplasm. The uncropped blots are shown in Supplementary File.

To identify the lncRNA HCP5-encoded peptides or proteins in breast cancer, we integratively analyzed the HCP5 open reading frame (ORF), ribosome profile (Ribo-seq), and MS/MS data of the MDA-MB-231 cell line. The workflow diagram of HCP5-encoded small peptides (sPEPs) identification is shown in Figure 1A. The LncRNA HCP5 sequence (GRCh38 fasta format) was obtained from NCBI Gene [19]. Ribo-seq data (GSE69923) and MS/MS data (PXD008222) were downloaded from the Gene Expression Omnibus (GEO) database [20] and EMBI-EBI-PRIDE database [21], respectively.

HCP5 ORFs were identified by ORFfinder [22] in NCBI based on HCP5 exon sequence. We screened ORFs with start codon “ATG” in the positive strand as candidates. Then, we obtained Ribo-seq files of SRA format and converted them to fastq format by using the fastq-dump tool (https://ncbi.github.io/sra-tools/fastq-dump.html, accessed on 10 January 2019). Next, adaptor sequences were trimmed using Trim Galore (https://www.bioinformatics.babraham.ac.uk/projects/trim_galore/, accessed on 10 January 2019). Reads shorter than 25 bp after adaptor trimming were discarded. rRNA sequences were filtered by using the RNAcentral database [23]. Furthermore, the remaining reads were aligned to reference genome GRCh38 using TopHat2 [24] and bam format file was obtained. The ORFs fewer than 400 bp with reads mapped were regarded as convinced HCP5 small ORFs (sORFs). In addition, MS/MS raw data were converted to mgf format by MSConvert [25]. Then, Peppy was used to get amino acid (aa) sequences for peptides or proteins and aligned to GRCh38. Finally, the convinced HCP5 sORFs with MS/MS peptides mapped were validated as highly convinced HCP5 sORFs.

### 2.2. Tissue Samples

Breast tumor tissue microarrays (TMA) were obtained from Shanghai Outdo Biotech Co. (Shanghai, China). The TMA HBre-Duc140Sur-01 contains 140 cases of invasive ductal carcinomas and 45 pared precancerous breast tissues from the regions around cancers. No patients received adjuvant radiotherapy, chemotherapy, or immunotherapy before surgery. The experimental protocols were approved by The Human Research Ethics Committee from Medical College of Southeast University.

### 2.3. Immunohistochemistry Staining (IHC)

The tissue sections were dried at 60 °C for 1 h, then dewaxed in xylene and rehydrated through graded alcohol concentrations using standard procedures. Antigen retrieval was performed in citrate buffer (pH 6.0) and autoclave at 121 °C for 90 s. After washing in PBS (3 min × 3), sections were blocked with goat serum (Boster, Wuhan, China) at room temperature for 30 min. Then, each section was treated with HCP5-132aa mouse polyclonal antibodies (H00010866-B01P, Abnova, Inc., Taiwan, China), at a dilution of 1:200 solution) at 4 °C overnight. After washing in PBS (5 min × 3), each section was incubated with a Polink-1 HRP DAB Detection System One-step polymer detection system for mouse antibody (ZSGB-BIO, Beijing, China) at room temperature for 20 min. After washing in PBS (3 min × 3), the slides were counterstained with hematoxylin. For negative controls, the primary antibody was substituted with PBS.

### 2.4. Reagents and Cell Viability Assay

Ferrostatin-1 (Fer-1; SML0583) was obtained from Sigma-Aldrich, Liproxstatin-1 (Lip-1; S7699), and RLS3 (S8155) and Erastin (S7242) were obtained from Selleck. cn. MDA-MB-231 cells (5 × 10^4^/mL) were seeded in 96-well plates with 100 μL per well for 24 h, then 0.099 μM, 0.197 μM, 0.375 μM, 0.625 μM, 1.25 μM, 2.5 μM, 5 μM, 10 μM, and 20 μM RLS3, Sulfasalazine and Erastin were added into plates. After 16 h, 24 h and 48 h, 10 μL of the Cell Counting Kit-8 (CCK-8) reagent was added into each well and incubated at 37 °C, 5% CO_2_ for 2 h. Then, cell growth was detected, and the inhibition rate was calculated.

### 2.5. Cell Culture and Lentivirus-Mediated Transduction of shRNA

All cell lines were purchased from National Collection of Authenticated Cell Cultures. Human breast cancer cell lines MDA-MB-231 and MDA-MB-468 were cultured in L-15 medium with 10% fetal bovine serum (FBS), at 37 °C in an incubator without CO_2_. Normal human mammary epithelial cells MAF-10A were cultured in medium (CM-0525, Procell. com.cn, Wuhan, China) at 37 °C in an incubator with CO_2_.

The procedure of lentivirus infection is as follows: the plate containing cells was added with the appropriate amount of lentivirus in concentration gradient, followed by adding 1/1000 polybrene to enhance infection. The sequence of the HCP5-132aa shRNA is shown in Table S1. Lentivirus vector LV5 containing empty vector, full-length HCP5-132aa ORF or HCP5-132aa ORF-mut (lncRNA HCP5 with HCP5-132aa ORF start codon mutated to ATT) were purchased from GenePharma Co., Ltd. (Shanghai, China).

### 2.6. Cell Proliferation Assays

MDA-MB-231 cells, MDA-MB-468 cells, and MAF-10A cells were seeded in 96-well plates and transfected with HCP5-132aa, Vector, HCP5-132aa shRNA, and negative controls (NCs), respectively. After transfection for 48 h, 10 μL of the CCK-8 reagent was added into each well, incubated at 37 °C, 5% CO_2_ for 2 h, and cell growth was detected by an enzyme labeling instrument at 450 nm.

### 2.7. Colony-Formation Assays

For colony-formation assays with monolayer culture, MDA-MB-231 cells (0.4 × 10^3^/well) and MDA-MB-468 cells (0.4 × 10^3^/well) were plated in a 6-well plates for two weeks. After being fixed with methanol, the cells were stained with 0.1% crystal violet for 30 min and then the colonies were imaged and counted.

### 2.8. Transwell

For transwell migration, 1 × 10^5^/mL cells were suspended in 200 μL medium without bovine serum albumin into the upper chamber of 24-well transwell plates (8 μm pore size; Corning, NY, USA), and 500 μL of medium containing 10% FBS was added to the lower chambers. After 24 h of co-culture, the cells on the lower surface of the membrane were fixed in 4% paraformaldehyde, stained by 0.1% crystal violet. The stained cells were then counted under light microscope. Photographs of random fields across three replicate wells by 200 times magnification were captured for analysis.

### 2.9. RNA Extraction and Quantitative Reverse Transcription PCR

TRIzol (Invitrogen, Carlsbad, CA, USA) was used to isolate the total RNA of tissues, and cDNA was synthesized with PrimeScriptTM RT Master Mix (Takara Biomedical Technology Co., Ltd., Beijing, China). A qTOWER2.0 Real-Time PCR System (Analytik Jena AG, Jena, Germany) with ChamQ Universal SYBR qPCR Master Mix (Vazyme Biotech Co., Ltd., Nanjing, China) was used to perform quantitative PCR (qPCR). Relative mRNA expression was standardized using the housekeeping GAPDH. The following human primers purchased from Sangon Biotech (Shanghai, China) were as follows: HCP5-132aa R: 5′-GAGGCATGGCTGCTGTCACAC-3′, F: 5′-TGGCTGGACGATTCTCCTCACAC-3′; GAPDH R: 5′-TCTCGCTCCTGGAAGATGGTGAT-3′, F: 5′-CGGAGTCAACGGATTTGGTCG-3′. The procedures were performed in triplicate.

### 2.10. RNA Sequencing, Differential Expression Analysis, and Functional Enrichment Analysis

MDA-MB-231 cells were transfected with shNC and shHCP5-132aa lentivirus. Total amounts and integrity of RNA were assessed using the RNA Nano 6000 Assay Kit of the Bioanalyzer 2100 system (Agilent Technologies, Santa Clara, CA, USA). Then, library preparation for transcriptome sequencing was performed. After the library is qualified, the different libraries are pooling according to the effective concentration and the target amount of data off the machine, then being sequenced by the Illumina NovaSeq 6000. The RNA sequencing was performed by Novogene Co., Ltd. (Beijing, China).

To identify differentially expressed genes (DEGs) between the HCP5-132aa ORF knockdown and shNC samples, we used DEseq2 to perform differential expression analysis [26]. Genes with *q*-values < 0.05 and |log2 (Fold Change)| > log2 (1.5) were considered as significantly differentially expressed. Then, to perform functional enrichment analysis of DEGs, we utilized the *enrichKEGG* function of clusterProfiler R package [27]. Significant pathways were identified using a cutoff of *p* < 0.05.

### 2.11. Western Blot

MDA-MB-231 and MDA-MB-468 cells transfected with vector, HCP5-132aa, shHCP5-132aa, or shNC were harvested and lysed with cell lysis buffer for western blotting (Beyotime, Shanghai, China). The proteins (30 μg per lane) were separated on 12% SDS-polyacrylamide gels and transferred into polyvinylidene fluoride (PVDF) membranes (Millipore, Billerica, MA, USA). Primary antibodies for mouse-anti-AMID/AIFM2 (B-6) (1:500) (sc-377120, Santa Cruz Biotechnology, Inc., Dallas, TX, USA), mouse-anti-FTH1 (1:500) (sc-376594, Santa Cruz Biotechnology, Inc., Dallas, TX, USA), mouse-anti-ACSL4 (1:500) (sc-365230, Santa Cruz Biotechnology, Inc., Dallas, TX, USA), rabbit-anti-GPX4 (1:1000) (ab125066, Abcam, Cambridge, MA, USA), HCP5-132aa (1:1500) (H00010866-B01P, Abnova, Taiwan, China), and rabbit-anti-β-actin (1:1000) (Biosynthesis Biotechnology Inc., Beijing, China) were incubated at 4 °C overnight. Binding of the primary antibody was detected using an enhanced chemiluminescence kit (ECL, Amersham, Bioscience, PA, USA). The Image J software (ImageJ 1.5, NIH, USA) was used to quantify and analyze each specific western blot band.

### 2.12. Lipid ROS Assays

Lipid ROS levels were determined using C11-BODIPY dye (D3861, Thermo Fisher Scientific Inc., Waltham, MA, USA), according to the manufacturer’s instructions. Cells were seeded in 6-well plates overnight, then the culture medium was replaced with Erastin (15 μM), RSL3 (1.25 μM), Liproxstatin-1 (50 μM), or Ferrostatin-1 (1 μM) treatment for 48 h. Then, the medium was replaced with 5 μM C11-BODIPY-containing medium for 1 h. Later, the cells were harvested by trypsin and washed three times with ice-cold PBS followed by re-suspending in PBS plus 1% BSA. The amount of ROS within cells was examined by flow cytometry analysis (BD FACSCantoTM II, NJ, USA).

### 2.13. Immunofluorescence Analysis

Cells were fixed in 4% formaldehyde for 30 min at room temperature before cell permeabilization with 0.1% Triton X-100 (4 °C, 10 min). Cells were saturated with PBS containing 2% bovine serum albumin for 1 h at room temperature and processed for immunofluorescence with 1 mg/mL C11-BODIPY followed by 10 mg/mL Hoechst 33,258 (Invitrogen, Waltham, MA, USA). Between all incubation steps, cells were washed three times for 3 min with PBS containing 0.2% bovine serum albumin. Fluorescence signals were analyzed using an Olympus Fluoview 1000 confocal microscope (Olympus Corp, Tokyo, Japan).

### 2.14. Transmission Electron Microscopy (TEM)

Cells were collected and fixed in 2.5% glutaraldehyde for at least 3 h. Then, the cells were treated with 2% paraformaldehyde at room temperature for 60 min and 0.1% glutaraldehyde in 0.1 M sodium cacodylate for 2 h, followed by post-fixing with 1% OsO_4_ for 1.5 h. After a second washing, cells were dehydrated with graded acetone and finally embedded in Quetol 812. Ultrathin sections were observed under an H7500 electron microscope (Hitachi, Tokyo, Japan).

### 2.15. Xenograft Assay in Nude Mice

For each experiment, 24 mice were randomly divided into the following four groups: (1) control shRNA model receiving DMSO (vehicle); (2) control shRNA model receiving Erastin; (3) HCP5-132aa shRNA model receiving DMSO (vehicle); and (4) HCP5-132aa shRNA model receiving Erastin. Indicated subcutaneously injected into the dorsal flanks right of the midline in nude mice (female, 4~6 weeks). At day seven, mice were intraperitoneally injected with Erastin (50 mg/kg i.v., two times a week) for three weeks. Erastin was dissolved in the vehicle (2% DMSO and 98% PBS) and prepared by Ultrasonic Cleaner (Fisher Scientific, Hampton, NH, USA). A final volume of 300 μL of Erastin was applied via intraperitoneal injection. Tumors were measured twice a week. The volumes were calculated using the following formula: volume (mm^3^) = length × width^2^ × π/6. All animal experiments were performed in the animal laboratory center of the Medical College of Southeast University and in accordance with the Guide for the Care and Use of Laboratory Animals published by the US National Institutes of Health (NIH publication number 85-23, revised 1996).

### 2.16. Statistical Analysis

All statistical analyses were performed using SPSS 19.0 software (IBM Corp, Armonk, NY, USA). The results are expressed as the mean ± standard deviation (SD). Group means were compared using Student’s *t*-test for independent data. All *p*-values are two-tailed, and *p* < 0.05 was considered to indicate statistical significance. The chi-square test was used to compare HCP5-132aa expression between breast cancer tissues and paired breast tissues and the association with clinicopathologic parameters. Survival analyses were estimated using the Kaplan–Meier method.

## 3. Results

### 3.1. LncRNA HCP5 Encoded a Protein and Upregulated in TNBC Cell Lines

HCP5 is an lncRNA gene initially located in chr6 (p21.33) of Homo sapiens, and our previous report showed that it promotes the progression of TNBC [18]. In this study, we used ORFfinder to predict the potential sORFs in the exons of HCP5. The workflow diagram of HCP5-encoded sPEPs identification is shown in Figure 1A. Three highly convinced sORFs were further confirmed by mapping Ribo-seq reads and MS/MS peptides (Figure S1). We named the peptides encoded by HCP5 sORFs as sPEPs, with lengths of 132aa, 43aa, and 22aa (Table S2). Moreover, we identified a 399 bp ORF with the potential to encode a 132aa protein, which we named HCP5-132aa (Figure 1B–D).

To determine whether HCP5-132aa was expressed endogenously, we conducted western blot analysis to detect HCP5-132aa protein expression in normal human mammary epithelial cells MCF-10A and breast cancer cell lines T47D, MCF-7, SKBR3, MDA-MB-231, MDA-MB-468, and HCC-1937 cells. The molecular weight of HCP5-132aa is about 14 KDa. The results indicated that HCP5-132aa protein expression was higher in TNBC cell lines (MDA-MB-231, MDA-MB-468, and HCC-1937 cells) than in other subtypes of breast cancer cell lines and MCF-10A (Figure 1E). We then performed a protein subcellular localization experiment and found that HCP5-132aa was expressed in both the nucleus and cytoplasm (Figure 1F).

### 3.2. HCP5-132aa High Expression Indicated a Poor Prognosis for Breast Cancer Patients

**Figure 2:**
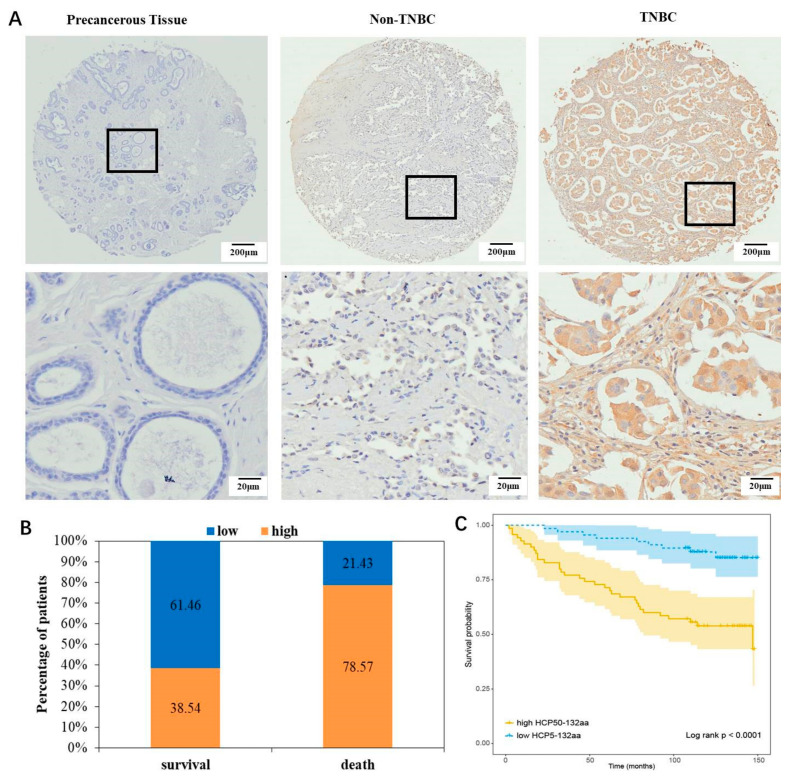
LncRNA HCP5-encoded protein upregulated in TNBC tissues. (**A**) Representative IHC images of HCP5-132aa protein expression in breast cancer tissues and their corresponding precancerous tissues. (**B**) Associations between HCP5-132aa protein levels (low or high) and the percentage of survival or death patients were analyzed in breast cancer samples. (**C**) Kaplan–Meier curves for overall survival of breast cancer patients with expression of HCP5-132aa. The breast cancer patients with high expression of HCP5-132aa showed worse overall survival rates (*p* < 0.0001).

To investigate the role of the HCP5-132aa in breast cancer patients, we analyzed protein levels in tissue microarray analysis of 140 breast cancer tissues and 39 matched precancerous tissues using IHC assay. The results showed that HCP5-132aa protein expression was higher in TNBC tissues than in non-TNBC and precancerous tissues (Figure 2A, Table 1 and Table 2, *p* = 0.042 and *p* < 0.001, respectively). Furthermore, increased levels of HCP5-132aa were positively associated with more advanced clinical stages of breast cancer (*p* = 0.002; Table 1). Kaplan–Meier survival analyses revealed that patients with higher HCP5-132aa levels had a higher risk of breast cancer-related death compared to patients with lower HCP5-132aa expression levels (Figure 2B,C, *p* < 0.0001, log-rank test). Therefore, increased HCP5-132aa protein expression was correlated with a poor prognosis in breast cancer patients.

### 3.3. HCP5-132aa Promoted TNBC Cell Malignant Phenotypes

**Figure 3:**
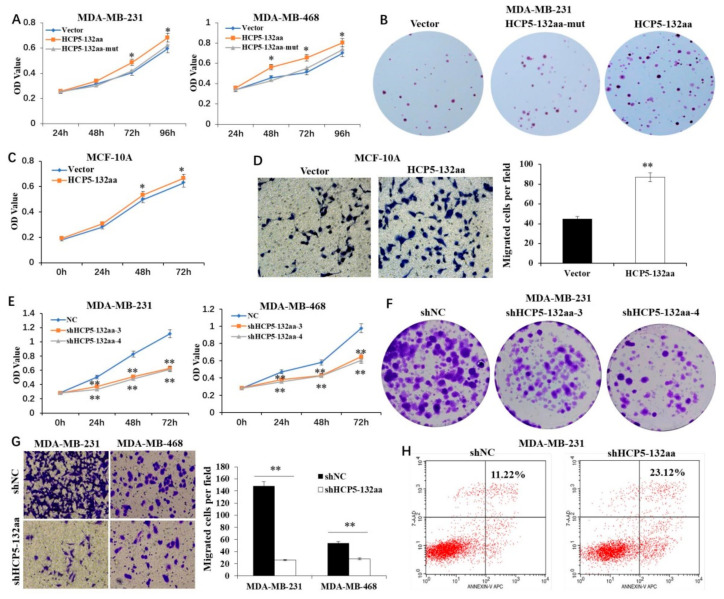
The HCP5-132aa promoted malignant phenotypes of TNBC cell lines. (**A**) MDA-MB-231 and MDA-MB-468 cells were transfected with the lentivirus vector, HCP5-132aa ORF or HCP5-132aa ORF-mut plasmids for the indicated times, and the cell numbers were measured by CCK8 (*n* = 3). (**B**) MDA-MB-231 cells were transfected with the indicated constructs, and their colony-forming abilities were measured after 2 weeks (×1). (**C**) MCF-10A cells were transfected with the vector or HCP5-132aa ORF plasmids for the indicated times, and the cell numbers were measured by CCK8 (*n* = 3). (**D**) MCF-10A cells were transfected with the vector or HCP5-132aa ORF plasmids, and migration abilities were determined using Transwell assays (×400). (**E**) MDA-MB-231 and MDA-MB-468 cells were transfected with the lentivirus negative control, shRNA-3, or shRNA-4 for the indicated times, and the cell numbers were measured by CCK8 (*n* = 3). (**F**) MDA-MB-231 cells were transfected with the indicated constructs, and their colony-forming abilities were measured after 2 weeks (×1). (**G**) MDA-MB-231 and MDA-MB-468 cells were transfected with the lentivirus negative control or shHCP5-132aa (shRNA-4), and migration abilities were determined using Transwell assays (×400). (**H**) MDA-MB-231 cells were transfected with the lentivirus negative control or shHCP5-132aa, and the percentage of apoptosis cells were measured by flow cytometry. * *p* < 0.05, ** *p* < 0.01.

To investigate the effects of the HCP5-132aa on TNBC cell progression, we constructed the overexpression HCP5-132aa ORF and HCP5-132aa ORF-mut plasmid. The HCP5-132aa ORF-mut plasmid contained a mutated ORF of lncRNA HCP5 to disrupt the encoded process. Our findings revealed that the overexpression of HCP5-132aa significantly promoted the growth of MDA-MB-231 and MDA-MB-468 cells, compared to the empty vector group. However, the mutation in HCP5-132aa ORF failed to show any such effects (Figure 3A). Moreover, the overexpression of HCP5-132aa, but not the mutation one, resulted in increased colony formation of MDA-MB-231 cells (Figure 3B). To determine if HCP5-132aa can transform normal human mammary epithelial cells into a more malignant phenotype, we conducted CCK8 and transwell experiments using MCF-10A cells. Our results indicated that HCP5-132aa overexpression can promote MCF-10A proliferation and migration (Figure 3C,D).

In our next set of experiments, we knocked down HCP5-132aa ORF using four shRNAs. We confirmed the knockdown and overexpression efficiency via q-PCR and western blot. Our findings showed that shHCP5-132aa-3 and shHCP5-132aa-4 had better knockdown efficiency (Figure S2A–D). We observed that both shHCP5-132aa-3 and shHCP5-132aa-4 inhibited cell growth of MDA-MB-231 and MDA-MB-468 cells (Figure 3E) and decreased colony formation of MDA-MB-231 cells (Figure 3F). We used shHCP5-132aa-4 to knock down HCP5-132aa ORF in our subsequent experiments. Downregulation of HCP5-132aa ORF significantly inhibited the migration ability of MDA-MB-231 and MDA-MB-468 cells (Figure 3G) and promoted MDA-MB-231 apoptosis (Figure 3H). Our results suggest that HCP5-132aa plays a significant role in promoting TNBC cells progression.

### 3.4. Knockdown of HCP5-132aa ORF-Promoted Ferroptosis

**Figure 4:**
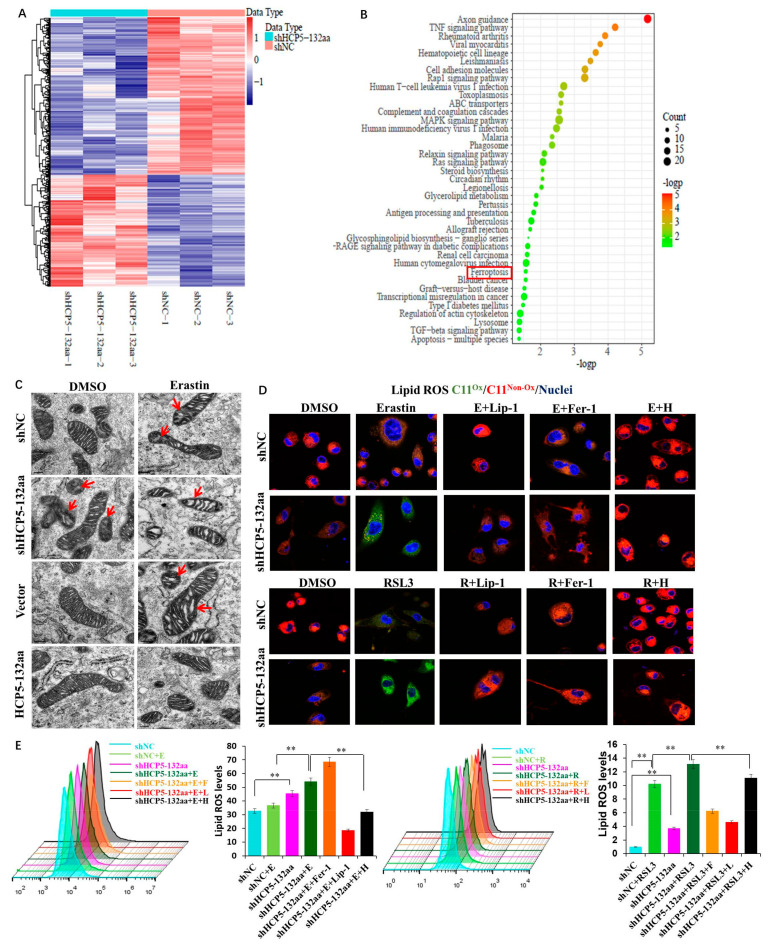
HCP5-132aa ORF knockdown promoted ferroptosis and increased ROS production. (**A**) The heatmap of DEGs between shNC and shHCP5-132aa MDA-MB-231 cells. (**B**) KEGG pathway enrichment analysis of the DEGs. The red square emphasizes the ferroptosis pathway. (**C**) Mitochondrial morphological changes stimulating with Erastin in HCP5-132aa ORF knockdown or overexpressed MDA-MB-231 cells were observed using TEM (red arrows show the reduced mitochondrial crests) (×8000). (**D**) MDA-MB-231 cells were treated with DMSO (D, 1 μM), Erastin (E, 15 μM), RSL3 (R, 1.25 μM), Liproxstatin-1 (Lip-1, 50 μM), Ferrostatin-1 (Fer-1, 1 μM) or HCP5-132aa ORF overexpression plasmid (H, 30 μL) for 48 h, respectively. Confocal images of C11 BODIPY 581/591 were obtained. Cells were labeled with C11 (5 μM) and Hoechst (1 μg/mL) prior to imaging. C11^Ox^: oxidizedC11; C11^Non-Ox^: non-oxidized C11 (×1000). (**E**) ROS generation was quantified by flow cytometry, as described in Section 2. Images were representative of three independent experiments. ** *p* < 0.01.

In order to verify the regulatory mechanism of HCP5-132aa in TNBC, we performed RNA sequencing to identify DEGs after knockdown of HCP5-132aa ORF. Using DEseq2 for differential expression analysis, we identified 720 DEGs (*q* < 0.05 and |log2 (Fold Change)| > log2 (1.5)) (Figure 4A). Next, we performed functional enrichment analysis for the DEGs using the clusterProfiler R package, which revealed that the DEGs were significantly enriched in 39 pathways, including ferroptosis (*p* < 0.05) (Figure 4B). Since ferroptosis is a new type of programmed cell death defined in recent years, and some research has suggested that ferroptosis may be involved in cancer development, we aimed to investigate whether HCP5-132aa regulates ferroptosis to promote the malignant phenotype of TNBC. To further investigate the role of HCP5-132aa in ferroptosis, we induced ferroptosis in MDA-MB-231 cells using the ferroptosis activators Erastin and RSL3. We observed mitochondrial morphological changes using transmission electron microscopy (TEM). Specifically, we found that knockdown of HCP5-132aa ORF increased the mitochondrial membrane density and reduced the mitochondrial crest, which was consistent with the damage induced by Erastin in the shNC group. In contrast, there was no significant change in mitochondrial morphology when HCP5-132aa was overexpressed in MDA-MB-231 cells treated with the same dose of Erastin (Figure 4C). These results suggested that HCP5-132aa may protect cells from Erastin-induced ferroptosis.

As ferroptosis is known to cause lipid ROS accumulation, we measured lipid ROS levels using C11-BODIPY staining by flow cytometry and confocal microscopy in cells with knockdown of HCP5-132aa ORF and treated with ferroptosis stimulators or inhibitors. We found that the lipid ROS level increased in HCP5-132aa ORF knockdown cells and were further elevated after treatment with Erastin or RSL3. In contrast, co-treatment with HCP5-132aa overexpression plasmid and ferroptosis inhibitors, Fer-1 and Lip-1, suppressed lipid ROS levels in response to Erastin or RSL3 (Figure 4D,E). Taken together, these findings suggest that downregulation of HCP5-132aa ORF promotes ferroptosis by inducing lipid ROS production.

**Figure 5:**
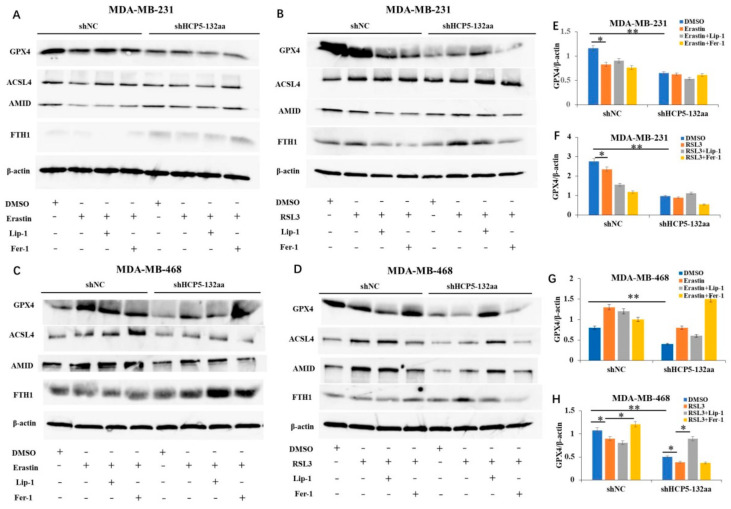
HCP5-132aa ORF knockdown affected the protein expression of ferroptosis pathways. (**A**–**D**) HCP5-132aa ORF knockdown or negative control MDA-MB-231 and MDA-MB-468 cells were lysed after treatment with DMSO, Erastin, RSL3, Liproxstatin-1 (Lip-1), or Ferrostatin-1 (Fer-1) for 48 h. Western blot determination of ferroptosis-related proteins GPX4, ACSL4, AMID, and FTH1 were performed. (**E**,**F**) Densitometry quantification of GPX4 was normalized to actin in MDA-MB-231 cells. (**G**,**H**) Densitometry quantification of GPX4 was normalized to actin in MDA-MB-468 cells. Images were representative of three independent experiments. ** *p* < 0.01, * *p* < 0.05. The uncropped blots are shown in Supplementary File.

We performed western blot analysis to investigate whether HCP5-132aa affects the protein expression of ferroptosis pathways Xc-/GSH/GPX4, ACSL4/LPCAT3/15-LOX, and AMID/CoQ10. Our results revealed that knockdown of the HCP5-132aa ORF led to a decrease in GPX4 protein expression in MDA-MB-231 cells. However, the expression levels of ACSL4, AMID, and FTH1 did not show significant changes. These findings suggest that HCP5-132aa may play a role in regulating the Xc-/GSH/GPX4 pathway of ferroptosis, no other ferroptosis pathways. When stimulated with ferroptosis activators Erastin or RSL3, the GPX4 expression was lower in the shNC group compared to the DMSO control, but there was no significant difference in the shHCP5-132aa group. The ferroptosis inhibitors Lip-1 and Fer-1 were unable to reverse the Erastin- or RSL3-induced GPX4 downregulation in both shNC and shHCP5-132aa groups, indicating that these inhibitors were not effective in MDA-MB-231 cells even after knockdown of the HCP5-132aa ORF (Figure 5A,B,E,F). In MDA-MB-468 cells, knockdown of the HCP5-132aa ORF resulted in a decrease in the GPX4 expression compared to shNC cells treated with DMSO, suggesting that knockdown of HCP5-132aa ORF could induce ferroptosis by regulating GPX4. However, there was no significant change in the expression of GPX4, ACSL4, AMID, and FTH1 when treated with Erastin compared to DMSO in both the shNC and shHCP5-132aa groups. These results suggest that Erastin cannot induce ferroptosis in MDA-MB-468 cells, whether HCP5-132aa is knocked down or not (Figure 5C,G). RSL3 stimulation resulted in GPX4 downregulation in both the shNC and shHCP5-132aa groups. Furthermore, Fer-1 and Lip-1 reversed the RSL3-induced GPX4 downregulation in the shNC and shHCP5-132aa groups, respectively (Figure 5D,H). Then, we investigated the expression of xCT, an upstream protein of GPX4, using western blot after knockdown of the HCP5-132aa ORF in both MDA-MB-231 and MDA-MB-468 cells. However, we observed no decrease in the expression of xCT protein (Figure S3). To further explore the role of HCP5-132aa in ferroptosis, we measured the GSH level in shNC and shHCP5-132aa MDA-MB-231 cells treated with DMSO, Erastin, or RSL3. Interestingly, we did not observe a decrease in the GSH concentration after knockdown of the HCP5-132aa ORF in these groups (Figure S4). These results suggest that knockdown of the HCP5-132aa ORF may specifically regulate GPX4 to induce ferroptosis in MDA-MB-231 and MDA-MB-468 cells, independent of the Xc-/GSH pathway.

### 3.5. HCP5-132aa Knockdown Inhibited Tumor Growth In Vivo

**Figure 6:**
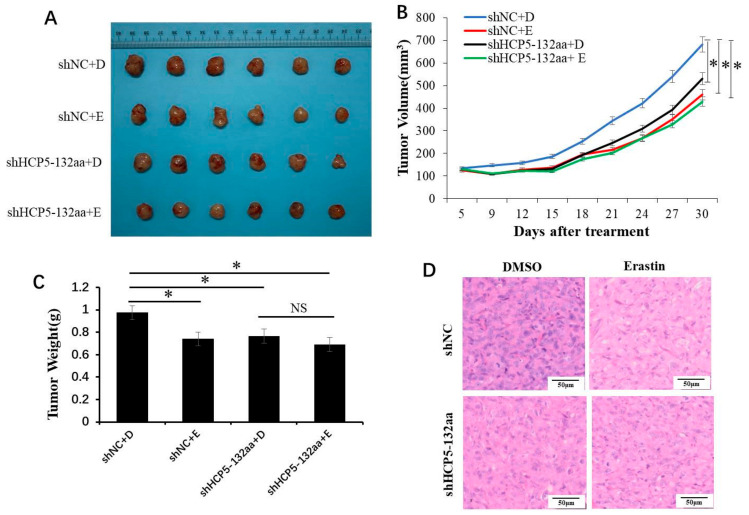
HCP5-132aa ORF knockdown inhibited TNBC cell growth in vivo. (**A**) Representative picture of tumor formation of xenograft in nude mice in shNC + DMSO, shNC + Erastin, shHCP5-132aa + DMSO and shHCP5-132aa + Erastin MDA-MB-231 cells (each group *n* = 6). (**B**) Summary of tumor volume of xenograft in nude mice. (**C**) Summary of tumor weight of xenograft in nude mice. (**D**) Representative HE stained tumor sections. * *p* < 0.05; NS, no significance.

To assess the effect of HCP5-132aa on TNBC in vivo, we treated subcutaneous xenograft tumors of nude mice with Erastin after silencing HCP5-132aa ORF. As shown in Figure 6A–C, Erastin injection and HCP5-132aa ORF knockdown inhibited tumor growth compared to the shNC + DMSO group in vivo. However, HCP5-132aa ORF knockdown did not synergize with Erastin to significantly inhibited tumor growth, consistent with the results in vitro. The tumor weight in the shNC + DMSO group was significantly higher than that in the other three groups. Hematoxylin and eosin (HE) stained tissue sections showed no damage to organs (lungs, livers, kidneys, and hearts) at the given dose (Figure S5). The reduction of tumor cells was more noticeable in the shHCP5-132aa + DMSO group and Erastin treatment groups than in the shNC + DMSO group (Figure 6D). These data suggest that HCP5-132aa downregulation can inhibit tumor growth in vivo, but it does not synergize with ferroptosis activators.

## 4. Discussion

Several studies have demonstrated that the expression of lncRNA HCP5 is upregulated in glioma, cervical cancer, and follicular thyroid cancer [28,29,30], but decreased in ovarian cancer [31]. In our previous publication, we indicated that lncRNA HCP5 acts as a ceRNA to regulate BIRC3 by sponging miR-219a-5p, promoting the progression of TNBC [18].

In this study, we provide the evidence that lncRNA HCP5 encodes a 132aa protein, which we named HCP5-132aa. Actually, there were research studies that reported that lncRNA HCP5 has an ORF that may code for a peptide of 132 aa (Q6M2N7.1), although the function was not verified [32]. For the first time, we indicate that HCP5-132aa can promote the malignant progression of TNBC by regulating the ferroptosis pathway both in vitro and in vivo. Specifically, these effects are dependent on GPX4 and lipid ROS levels, rather than the xCT/GSH pathway and other ferroptosis-associated pathways. The levels of HCP5-132aa were found to be increased in TNBC cell lines and primary cancer tissues, compared to their corresponding parental cell lines and precancerous tissues, respectively. Moreover, high expression of the HCP5-132aa was associated with poorer patient prognoses, indicating that HCP5-132aa may have the potential to serve as a prognostic factor for TNBC.

Recent advances in bioinformatics and biochemical methodologies have uncovered the potential of lncRNAs to encode concealed peptides or proteins, but despite these findings, only a small number of them have been functionally verified and characterized [16]. Furthermore, there have been limited reports on the role of lncRNA-encoded proteins/peptides in cancer progression, with only a few articles addressing this topic [33]. In this study, we identified and characterized the function of a protein encoded by the lncRNA HCP5 during tumorigenesis. Our findings demonstrate that HCP5-132aa promotes TNBC cell growth, colony formation, and migration, while also suppressing cell apoptosis. Notably, the HCP5-132aa ORF-mut plasmid failed to induce these effects, providing further evidence that HCP5-132aa functions as a tumor promotor.

Our RNA sequencing results revealed that knocking down the HCP5-132aa ORF led to DEGs that were enriched in the ferroptosis pathway. Ferroptosis, a recently discovered form of programmed necrosis, is characterized by the lethal accumulation of iron-dependent lipid ROS and is independent of apoptosis [34]. Ferroptotic cells typically exhibit smaller mitochondria, reduced or absent mitochondria crista, and condensed mitochondrial membrane densities [6]. We observed that knockdown of HCP5-132aa ORF alone induced ferroptosis, suggesting that it may act as a driver of this process. We are the first to propose that the protein encoded by an lncRNA can promote cancer development by regulating ferroptosis. We found that knocking down the HCP5-132aa ORF increased mitochondrial membrane density and reduced mitochondrial crest, similar to the effects of Erastin, while HCP5-132aa ORF overexpression suppressed the morphological changes in mitochondria induced by Erastin. We also showed that HCP5-132aa knockdown directly increased ROS levels, with the highest ROS levels observed when cells were stimulated with ferroptosis activators Erastin and RSL3. This effect could be reversed by ferroptosis inhibitors and HCP5-132aa overexpression. GPX4 is the only peroxidase known to efficiently reduce esterified, hydroperoxy fatty acids to unreactive alcohols, while ferroptosis suppressor protein 1 (FSP1), also known as AMID, was recently identified as a second ferroptosis suppression mechanism through its recycling of coenzyme Q10, a radical-trapping antioxidant [35]. However, western blot analysis showed that only GPX4 decreased after knockdown of HCP5-132aa ORF, while expression of the GSH level, xCT protein, and other ferroptosis pathway proteins ACSL4, AMID, and FTH1 were unchanged in MDA-MB-231 and MDA-MB-468 cells. We speculated that HCP50-132aa could activate GPX4 to promote the antioxidant response and decrease the accumulation of natural lipid ROS species, thereby promoting TNBC progression. Interestingly, lipid ROS accumulation was highest when the HCP5-132aa ORF was knocked down and cells were stimulated with Erastin or RSL3, but the GPX4 expression did not synergize with Erastin or RSL3 in shHCP5-132aa cells. These results suggested that there might be other pathways that induce lipid ROS accumulation in addition to ferroptosis after knockdown of the HCP5-132aa ORF. Our xenograft results also showed that knockdown of the HCP5-132aa ORF inhibited tumor growth in vivo, similar to the effects of Erastin injection.

## 5. Conclusions

**Figure 7:**
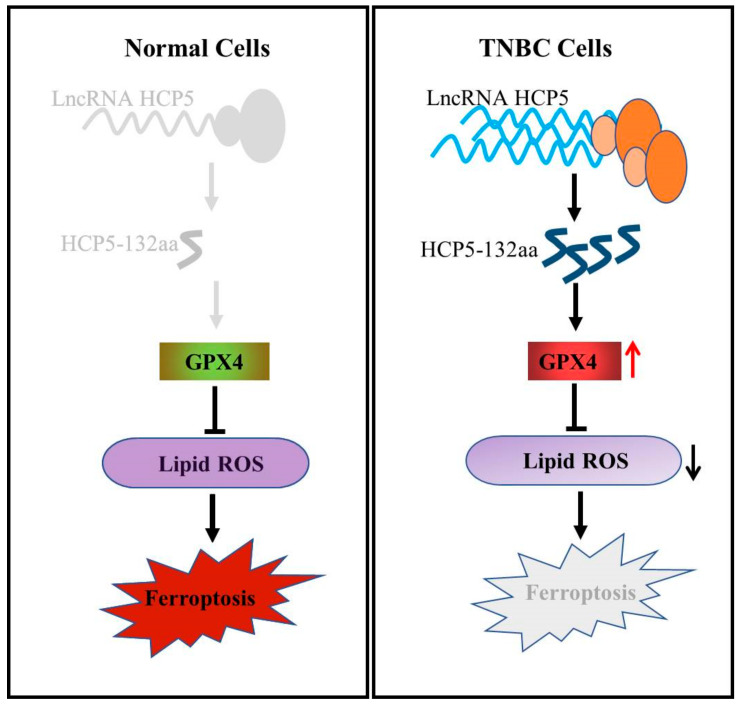
The schematic diagram of lncRNA HCP5-encoded protein inhibiting cell death through ferroptosis.

In summary, our study identified a novel protein HCP5-132aa encoded by the lncRNA HCP5, which promotes TNBC growth by regulating GPX4 and subsequently inhibiting ROS levels, thereby suppressing ferroptosis. We depicted the findings in Figure 7, and our analysis showed that TNBC patients with high expression of HCP5-132aa tend to have more aggressive clinicopathological features and a poorer prognosis. These findings significantly contribute to our understanding of the pathways that regulate ferroptosis and provide a promising therapeutic strategy for TNBC treatment.

## Figures and Tables

**Table 1:** Association between HCP5-132aa expression with clinicopathologic features of breast cancer patients.

	Low	High	χ^2^	*p*
Subtypes				
TNBC	4	12	4.143	0.042
Other subtypes	63	58		
TNM stage				
I, II	52	38	10.029	0.002
III	13	32		
LN metastasis				
−	28	20	3.231	0.072
+	34	47		
Tumor size				
<5 cm	57	55	1.984	0.159
≥5 cm	8	15		

**Table 2:** The expression of HCP5-132aa in paired breast cancer tissues.

	HCP5-132aa		*p*
	Low	High	
Precancerous tissues	39	0	<0.001
TNBC tissues	4	12	

## Data Availability

The data can be shared up on request.

## Supplementary Materials

The following supporting information can be downloaded at: https://www.mdpi.com/article/10.3390/cancers15061880/s1, Figure S1: Prediction results of HCP5-encoded small peptides; Figure S2: The knockdown or overexpressed HCP5-132aa ORF expression was identified in TNBC cells; Figure S3: Western blot determined the expression of ferroptosis-related proteins after HCP5-132aa ORF knockdown in TNBC cells. Figure S4: GSH levels were detected in HCP5-132aa ORF knockdown or negative control MDA-MB-231 cells. Figure S5: HE stained of lungs, livers, kidneys and hearts in nude mice. Table S1: HCP5-132aa ORF siRNA sequence; Table S2: Information of identified sPEPs. Supplementary File: The uncropped blots of Figures 1E,F, 5A–D and S3.

## References

[1] Ferlay J., Colombet M., Soerjomataram I., Parkin D.M., Pineros M., Znaor A., Bray F. (2021). Cancer statistics for the year 2020: An overview. Int. J. Cancer.

[2] Jemal A., Siegel R., Ward E., Hao Y., Xu J., Thun M.J. (2009). Cancer statistics, 2009. CA Cancer J. Clin..

[3] Dixon S.J., Lemberg K.M., Lamprecht M.R., Skouta R., Zaitsev E.M., Gleason C.E., Patel D.N., Bauer A.J., Cantley A.M., Yang W.S. (2012). Ferroptosis: An iron-dependent form of nonapoptotic cell death. Cell.

[4] Stockwell B.R., Friedmann Angeli J.P., Bayir H., Bush A.I., Conrad M., Dixon S.J., Fulda S., Gascon S., Hatzios S.K., Kagan V.E. (2017). Ferroptosis: A Regulated Cell Death Nexus Linking Metabolism, Redox Biology, and Disease. Cell.

[5] Xie Y., Hou W., Song X., Yu Y., Huang J., Sun X., Kang R., Tang D. (2016). Ferroptosis: Process and function. Cell Death Differ..

[6] Li D., Li Y. (2020). The interaction between ferroptosis and lipid metabolism in cancer. Signal Transduct. Target. Ther..

[7] Louandre C., Marcq I., Bouhlal H., Lachaier E., Godin C., Saidak Z., Francois C., Chatelain D., Debuysscher V., Barbare J.C. (2015). The retinoblastoma (Rb) protein regulates ferroptosis induced by sorafenib in human hepatocellular carcinoma cells. Cancer Lett..

[8] Wu J., Minikes A.M., Gao M., Bian H., Li Y., Stockwell B.R., Chen Z.N., Jiang X. (2019). Intercellular interaction dictates cancer cell ferroptosis via NF2-YAP signalling. Nature.

[9] Hu X., Sood A.K., Dang C.V., Zhang L. (2018). The role of long noncoding RNAs in cancer: The dark matter matters. Curr. Opin. Genet. Dev..

[10] Kung J.T., Colognori D., Lee J.T. (2013). Long noncoding RNAs: Past, present, and future. Genetics.

[11] Sun T. (2018). Long noncoding RNAs act as regulators of autophagy in cancer. Pharmacol. Res..

[12] Zhu S., Wang J., He Y., Meng N., Yan G.R. (2018). Peptides/Proteins Encoded by Non-coding RNA: A Novel Resource Bank for Drug Targets and Biomarkers. Front. Pharmacol..

[13] Wu P., Mo Y., Peng M., Tang T., Zhong Y., Deng X., Xiong F., Guo C., Wu X., Li Y. (2020). Emerging role of tumor-related functional peptides encoded by lncRNA and circRNA. Mol. Cancer.

[14] Wang Y., Wu S., Zhu X., Zhang L., Deng J., Li F., Guo B., Zhang S., Wu R., Zhang Z. (2020). LncRNA-encoded polypeptide ASRPS inhibits triple-negative breast cancer angiogenesis. J. Exp. Med..

[15] Huang J.Z., Chen M., Chen D., Gao X.C., Zhu S., Huang H., Hu M., Zhu H., Yan G.R. (2017). A Peptide Encoded by a Putative lncRNA HOXB-AS3 Suppresses Colon Cancer Growth. Mol. Cell.

[16] Xing J., Liu H., Jiang W., Wang L. (2020). LncRNA-Encoded Peptide: Functions and Predicting Methods. Front. Oncol..

[17] Tang C., Zhou Y., Sun W., Hu H., Liu Y., Chen L., Ou F., Zeng S., Lin N., Yu L. (2022). Oncopeptide MBOP Encoded by LINC01234 Promotes Colorectal Cancer through MAPK Signaling Pathway. Cancers.

[18] Wang L., Luan T., Zhou S., Lin J., Yang Y., Liu W., Tong X., Jiang W. (2019). LncRNA HCP5 promotes triple negative breast cancer progression as a ceRNA to regulate BIRC3 by sponging miR-219a-5p. Cancer Med..

[19] Morel A., O’Carroll A.M., Brownstein M.J., Lolait S.J. (1992). Molecular cloning and expression of a rat V1a arginine vasopressin receptor. Nature.

[20] Barrett T., Wilhite S.E., Ledoux P., Evangelista C., Kim I.F., Tomashevsky M., Marshall K.A., Phillippy K.H., Sherman P.M., Holko M. (2013). NCBI GEO: Archive for functional genomics data sets--update. Nucleic Acids Res..

[21] Vizcaino J.A., Csordas A., del-Toro N., Dianes J.A., Griss J., Lavidas I., Mayer G., Perez-Riverol Y., Reisinger F., Ternent T. (2016). 2016 update of the PRIDE database and its related tools. Nucleic Acids Res..

[22] Sayers E.W., Barrett T., Benson D.A., Bolton E., Bryant S.H., Canese K., Chetvernin V., Church D.M., DiCuccio M., Federhen S. (2011). Database resources of the National Center for Biotechnology Information. Nucleic Acids Res..

[23] The R.C. (2019). RNAcentral: A hub of information for non-coding RNA sequences. Nucleic Acids Res..

[24] Kim D., Pertea G., Trapnell C., Pimentel H., Kelley R., Salzberg S.L. (2013). TopHat2: Accurate alignment of transcriptomes in the presence of insertions, deletions and gene fusions. Genome Biol..

[25] Adusumilli R., Mallick P. (2017). Data Conversion with ProteoWizard msConvert. Methods Mol. Biol..

[26] Love M.I., Huber W., Anders S. (2014). Moderated estimation of fold change and dispersion for RNA-seq data with DESeq2. Genome Biol..

[27] Yu G., Wang L.G., Han Y., He Q.Y. (2012). clusterProfiler: An R package for comparing biological themes among gene clusters. OMICS.

[28] Teng H., Wang P., Xue Y., Liu X., Ma J., Cai H., Xi Z., Li Z., Liu Y. (2016). Role of HCP5-miR-139-RUNX1 Feedback Loop in Regulating Malignant Behavior of Glioma Cells. Mol. Ther. J. Am. Soc. Gene Ther..

[29] Yu Y., Shen H.M., Fang D.M., Meng Q.J., Xin Y.H. (2018). LncRNA HCP5 promotes the development of cervical cancer by regulating MACC1 via suppression of microRNA-15a. Eur. Rev. Med. Pharmacol. Sci..

[30] Liang L., Xu J., Wang M., Xu G., Zhang N., Wang G., Zhao Y. (2018). LncRNA HCP5 promotes follicular thyroid carcinoma progression via miRNAs sponge. Cell Death Dis..

[31] Liu N., Zhang R., Zhao X., Su J., Bian X., Ni J., Yue Y., Cai Y., Jin J. (2013). A potential diagnostic marker for ovarian cancer: Involvement of the histone acetyltransferase, human males absent on the first. Oncol. Lett..

[32] Kulski J.K. (2019). Long Noncoding RNA HCP5, a Hybrid HLA Class I Endogenous Retroviral Gene: Structure, Expression, and Disease Associations. Cells.

[33] Xiang X., Fu Y., Zhao K., Miao R., Zhang X., Ma X., Liu C., Zhang N., Qu K. (2021). Cellular senescence in hepatocellular carcinoma induced by a long non-coding RNA-encoded peptide PINT87aa by blocking FOXM1-mediated PHB2. Theranostics.

[34] Shaw A.T., Winslow M.M., Magendantz M., Ouyang C., Dowdle J., Subramanian A., Lewis T.A., Maglathin R.L., Tolliday N., Jacks T. (2011). Selective killing of K-ras mutant cancer cells by small molecule inducers of oxidative stress. Proc. Natl. Acad. Sci. USA.

[35] Bersuker K., Hendricks J.M., Li Z., Magtanong L., Ford B., Tang P.H., Roberts M.A., Tong B., Maimone T.J., Zoncu R. (2019). The CoQ oxidoreductase FSP1 acts parallel to GPX4 to inhibit ferroptosis. Nature.

