# Supplementary material for: LncRNA HCP5-Encoded Protein Regulates Ferroptosis to Promote the Progression of Triple-Negative Breast Cancer
Source: Cancers (Basel). 2023 Mar 21;15(6):1880. doi: 10.3390/cancers15061880 (PMC10046773; doi:10.3390/cancers15061880)
Supplement: Supplementary file 1 [file cancers-15-01880-s001.zip › cancers-2129240-supplementary-Figures and Tables.pdf]

Figure S1.

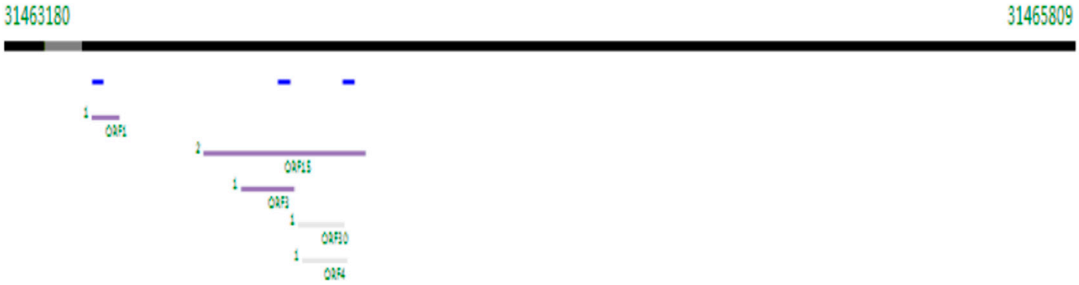

Figure S1. Prediction results of HCP5 encoded small peptides. Top line represents HCP5 sequence. Black region represents exon sequence. Grey region represents intron sequence. Intermediate blue short line represents Ribo-seq reads. Bottom line represents ORF and purple line represents ORF with MS/MS peptides mapped.

Figure S2.

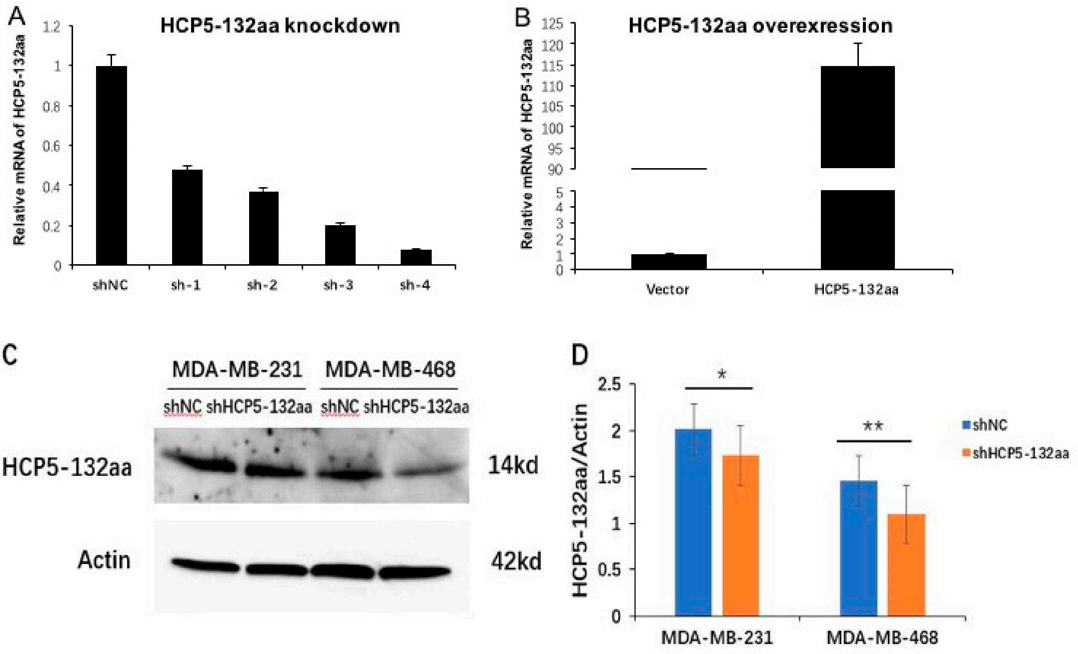

Figure S2. The knockdown or overexpressed HCP5-132aa ORF expression was identified in TNBC cells (A) The knockdown efficiency of HCP5-132aa in MDA-MB-231 cells was detected via q-PCR. (B) The overexpression efficiency of HCP5-132aa in MDA-MB-231 cells was detected via q-PCR. (C) The knockdown efficiency of HCP5-132aa was detected via western blot in MDA-MB-231 and MDA-MB-468 cells. (D) Densitometry quantification of GPX4 was normalized to actin in MDA-MB-231 cells. ** $P < 0.01$, * $P < 0.05$.

Figure S3.

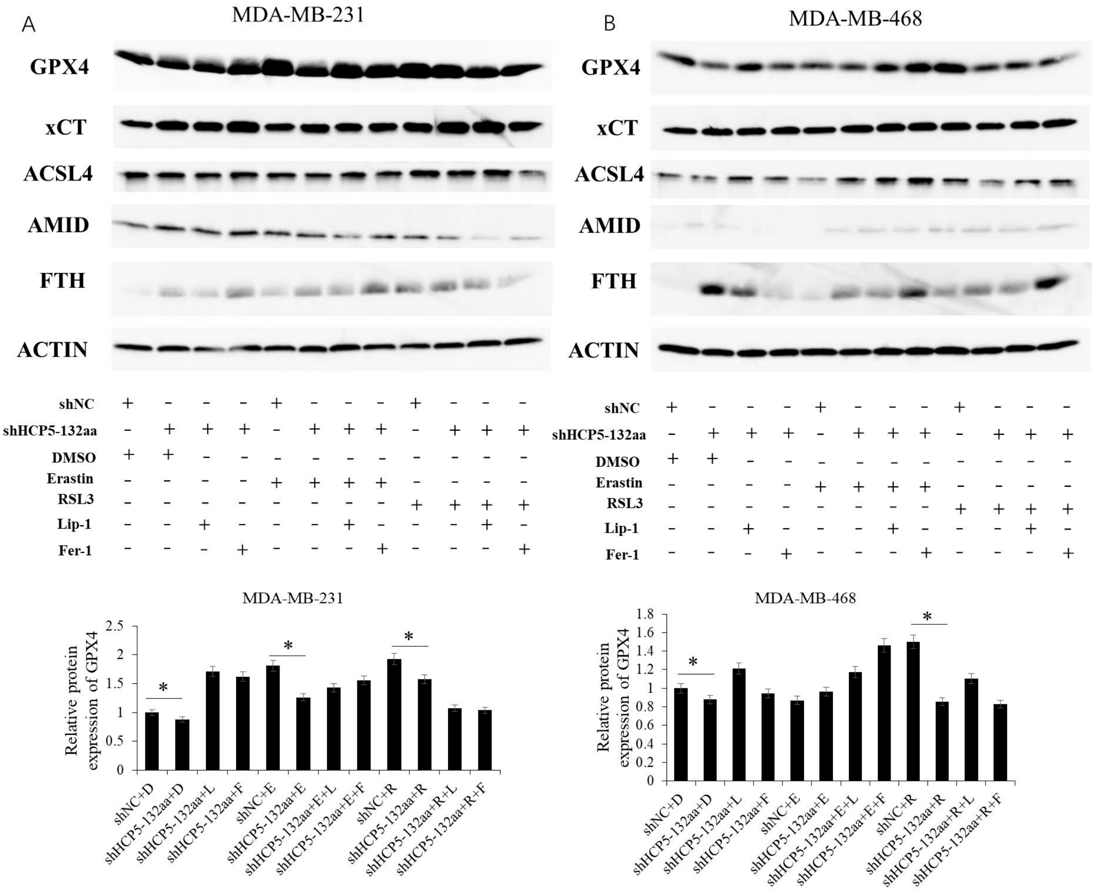

Figure S3. (A) HCP5-132aa ORF knockdown or negative control MDA-MB-231 cells were lysed after treatment with DMSO, Erastin, RSL3, Liproxstatin-1 (Lip-1) or Ferrostatin-1 (Fer-1) for 48 h. Western blot determination of ferroptosis-related proteins GPX4, xCT, ACSL4, AMID and FTH1 were performed. Densitometry quantification of GPX4 was normalized to actin in MDA-MB-231 cells. (B) HCP5-132aa ORF knockdown or negative control MDA-MB-468 cells were lysed after treatment with DMSO, Erastin, RSL3, Liproxstatin-1 (Lip-1) or Ferrostatin-1 (Fer-1) for 48 h. Western blot determination of ferroptosis-related proteins GPX4, xCT, ACSL4, AMID and FTH1 were performed. Densitometry quantification of GPX4 was normalized to actin in MDA-MB-468 cells. Images were representative of three independent experiments. * $P < 0.05$.

Figure S4.

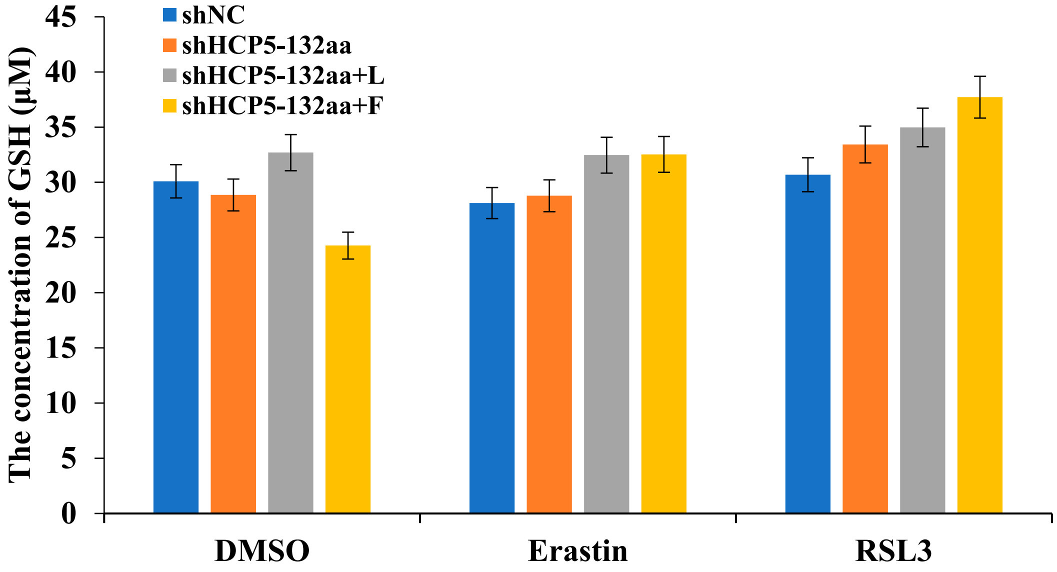

Figure S4. GSH levels were detected in HCP5-132aa ORF knockdown or negative control MDA-MB-231 cells treatment with DMSO, Erastin, RSL3, Fer-1 or Lip-1 for 48 h.

Figure S5

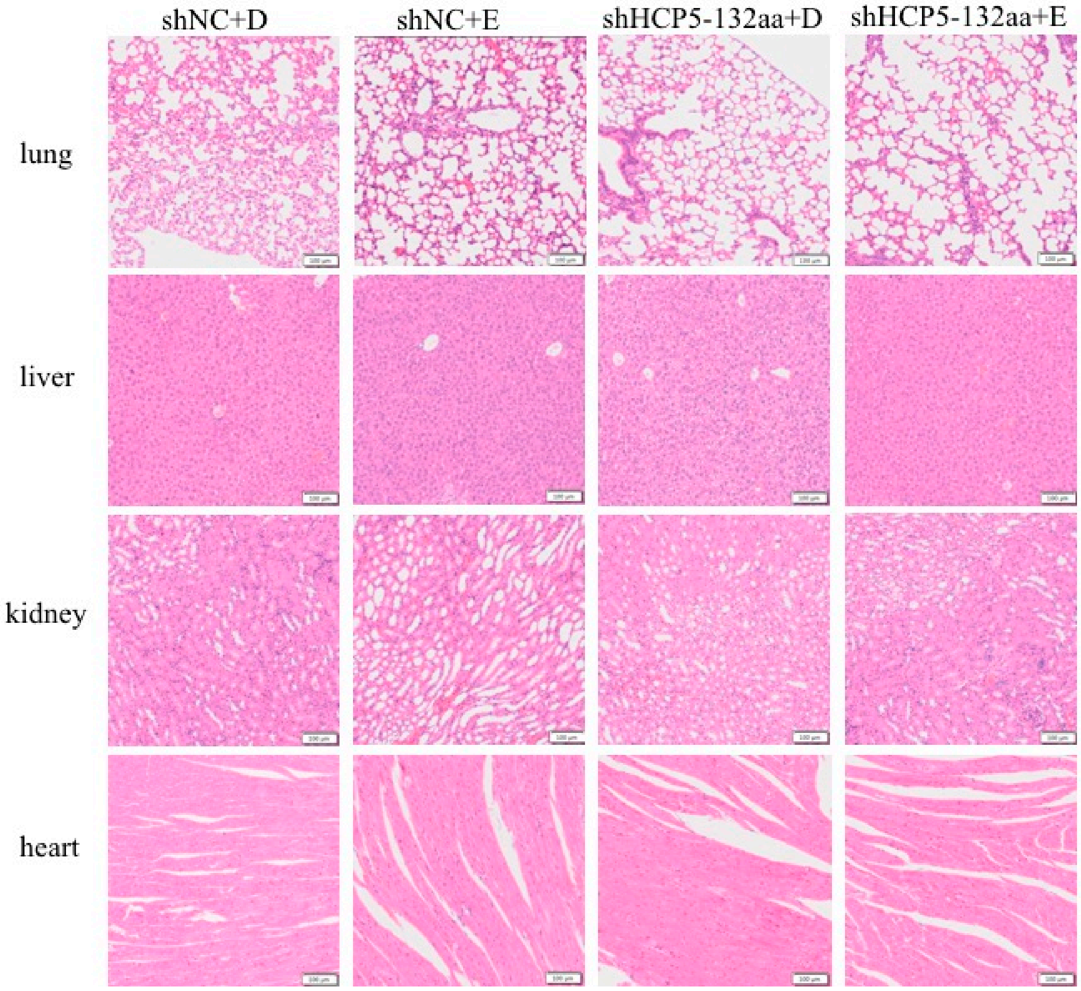

Figure S5. Hematoxylin and eosin (HE) stained tissue sections showed no damage to lungs, livers, kidneys and hearts of nude mice after DMSO or Erastin injection.

Table S1 HCP5-132aa ORF siRNA sequence

Gene name	5'to 3'
LV3-HCP5-homo-1	GAACTCCTCCTACCCTCATTG
LV3-HCP5-homo-2	GGTCTGGGCTCTTGGAAATCA
LV3-HCP5-homo-3	GGATCTATTACCTGTGCCTGG
LV3-HCP5-homo-4	GGACGATTCTCCTCACACTTA

Table S2 Information of identified sPEPs.

Name	Length	Sequence
sPEP 1	132aa	MLLRMSEHRNEALGNYLEMRLKSSFLRGLGSWKSNPLRLGGWTIL<br>LTLTMGQGEPGGPQGDPWVPHELLLPSLCDSSHASSWGSGSITCAW<br>RGGDSSSHPLVSGHILSNSPVAAVMCSSMGTHLSPFKGTLL
sPEP 2	43aa	MEIKPSQVGWLDDSPHTYNGTRGTRRPPRGSLGSTRTPPTLIV
sPEP 3	22aa	MISFLHYNSGKGNRLRCKCAPK
