# Supplementary material for: LncRNA HCP5-Encoded Protein Regulates Ferroptosis to Promote the Progression of Triple-Negative Breast Cancer
Source: Cancers (Basel). 2023 Mar 21;15(6):1880. doi: 10.3390/cancers15061880 (PMC10046773; doi:10.3390/cancers15061880)

Figure 1 E

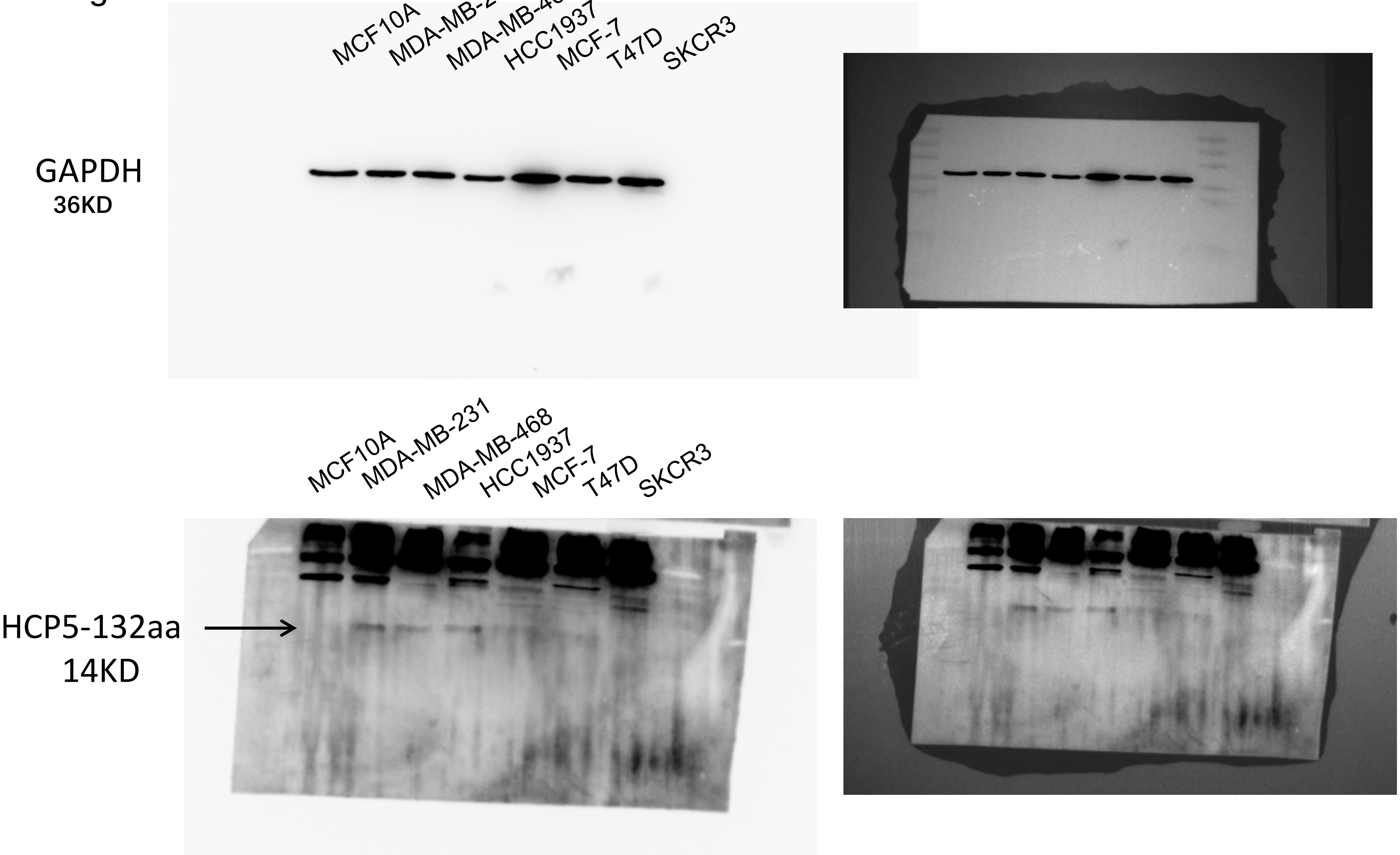
MCF10A
MDA-MB-231
MDA-MB-468
HCC1937
MCF-7
T47D
SKCR3
GAPDH
36KD
MCF10A
MDA-MB-231
MDA-MB-468
HCC1937
MCF-7
T47D
SKCR3
HCP5-132aa
14KD

Figure 1 F

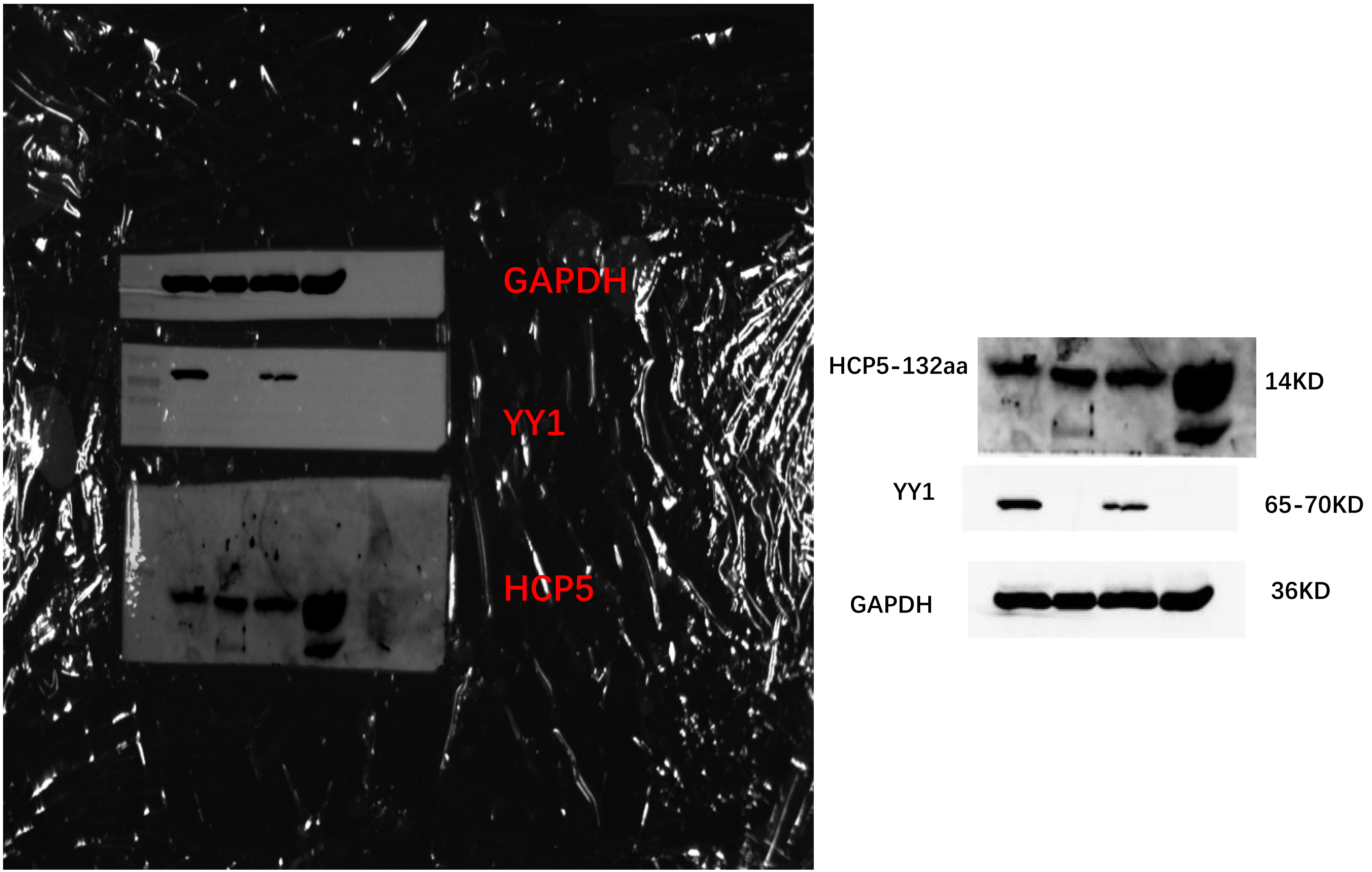

Figure 5A

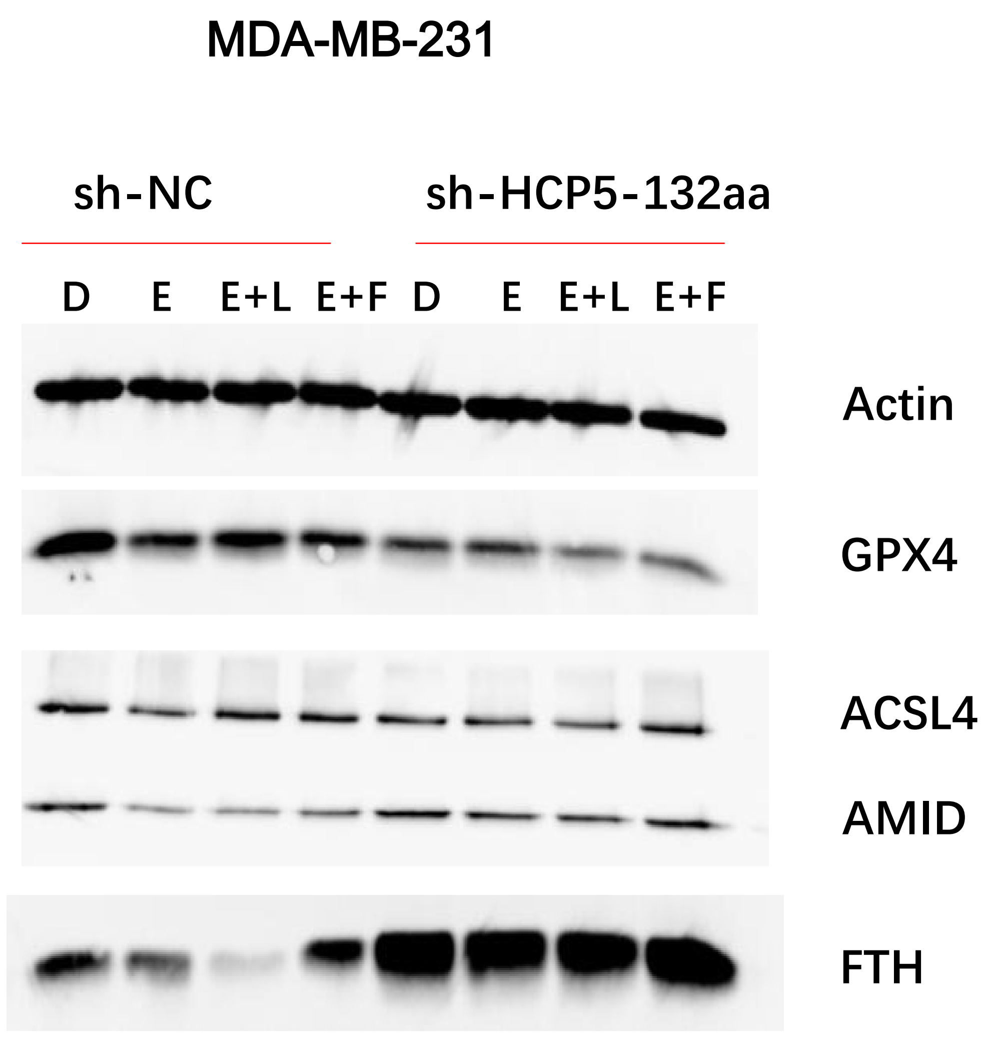
MDA-MB-231
sh-NC
sh-HCP5-132aa
D
E
E+L
E+F
D
E
E+L
E+F
Actin
GPX4
ACSL4
AMID
FTH

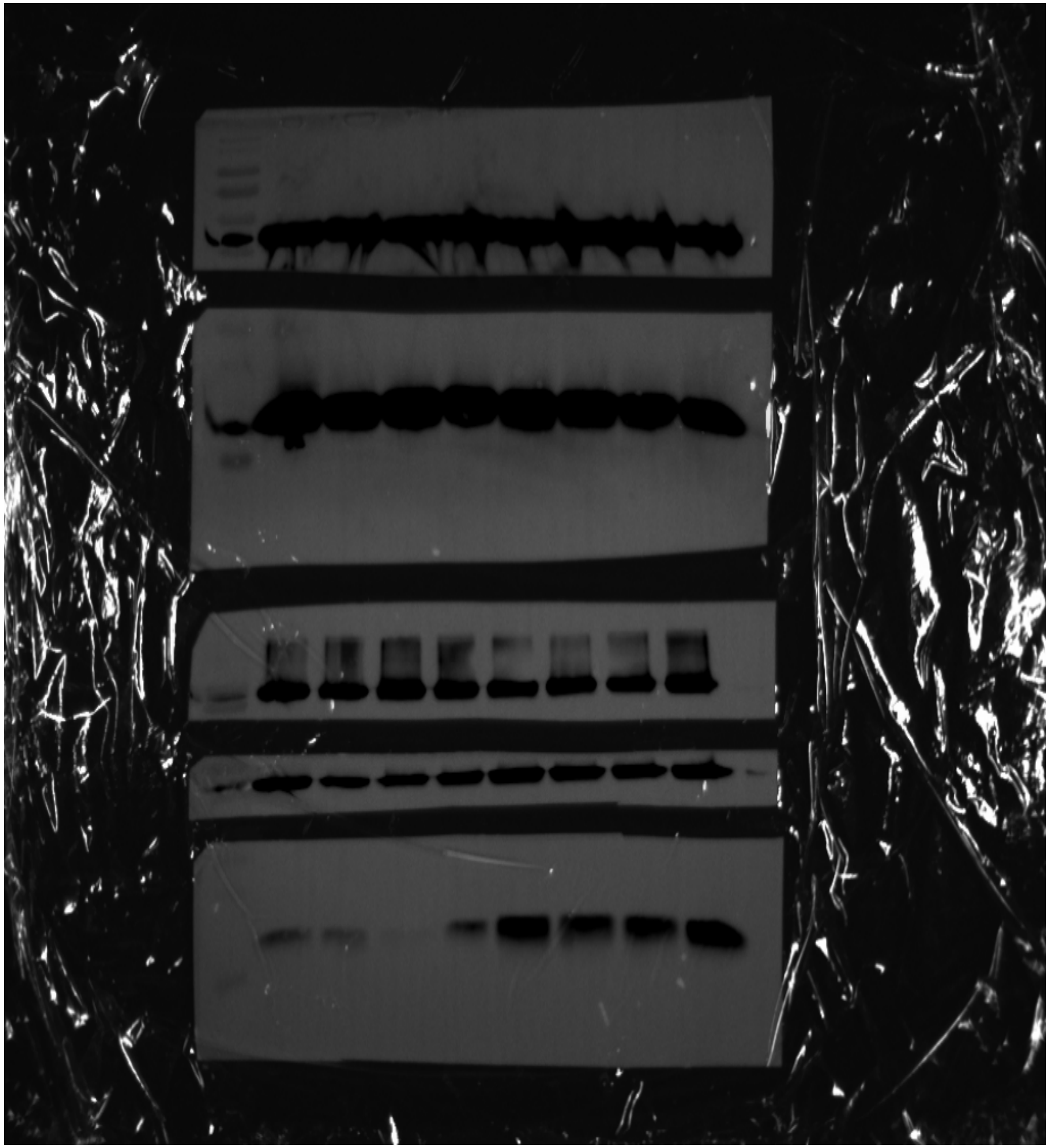

Actin

GPX4

ACSL4

AMID

FHC

Figure 5B

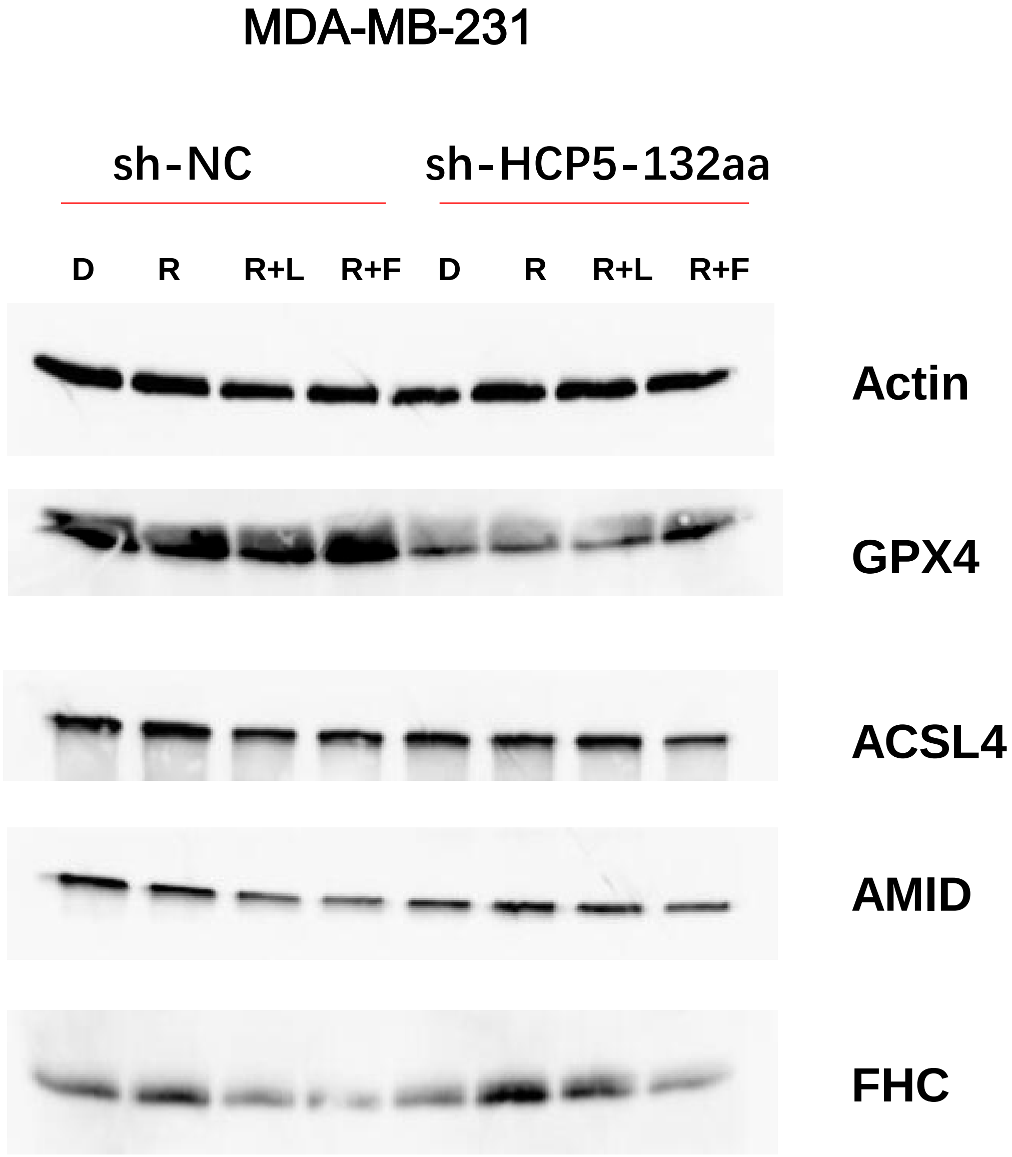
MDA-MB-231
sh-NC
sh-HCP5-132aa
D
R
R+L
R+F
D
R
R+L
R+F
Actin
GPX4
ACSL4
AMID
FHC

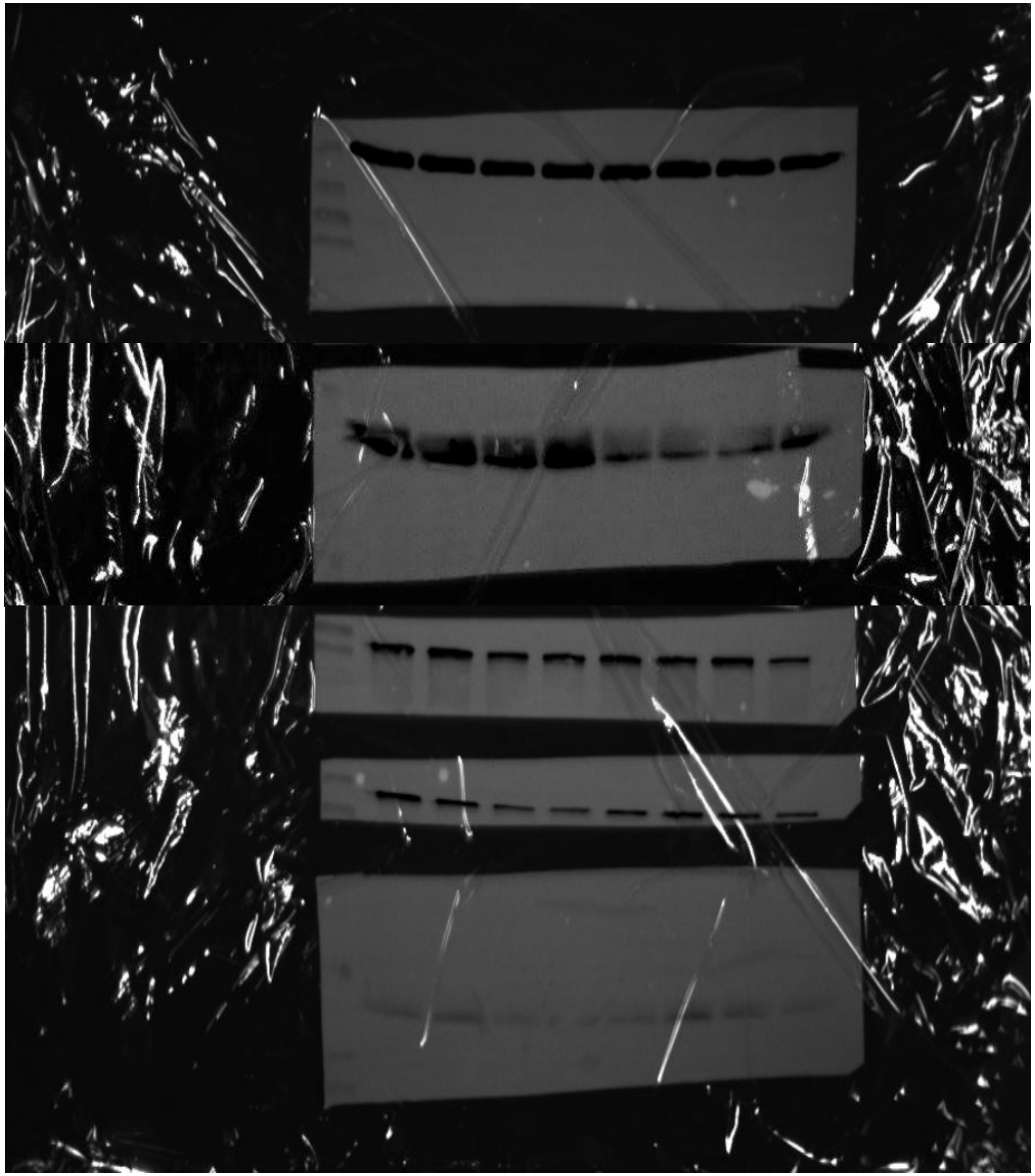

Actin

GPX4

ACSL4

AMID

FHC

Figure 5C

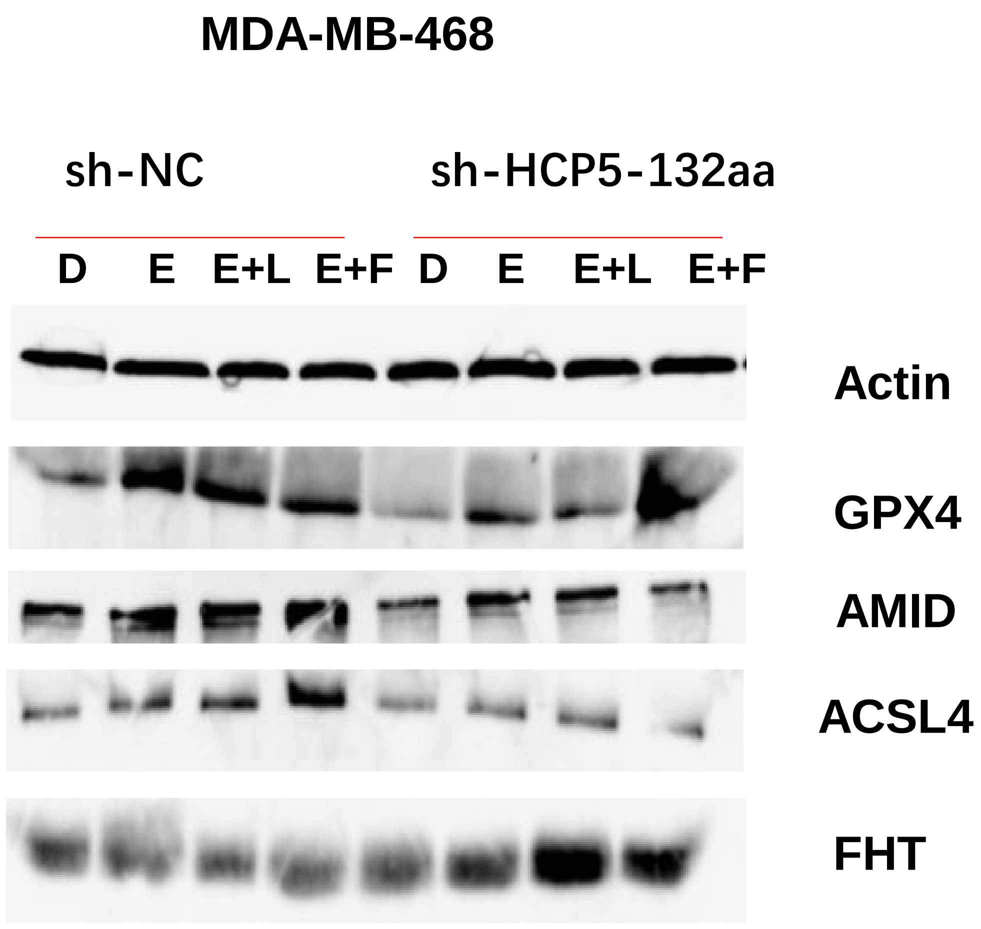
MDA-MB-468
sh-NC
sh-HCP5-132aa
D
E
E+L
E+F
D
E
E+L
E+F
Actin
GPX4
AMID
ACSL4
FHT

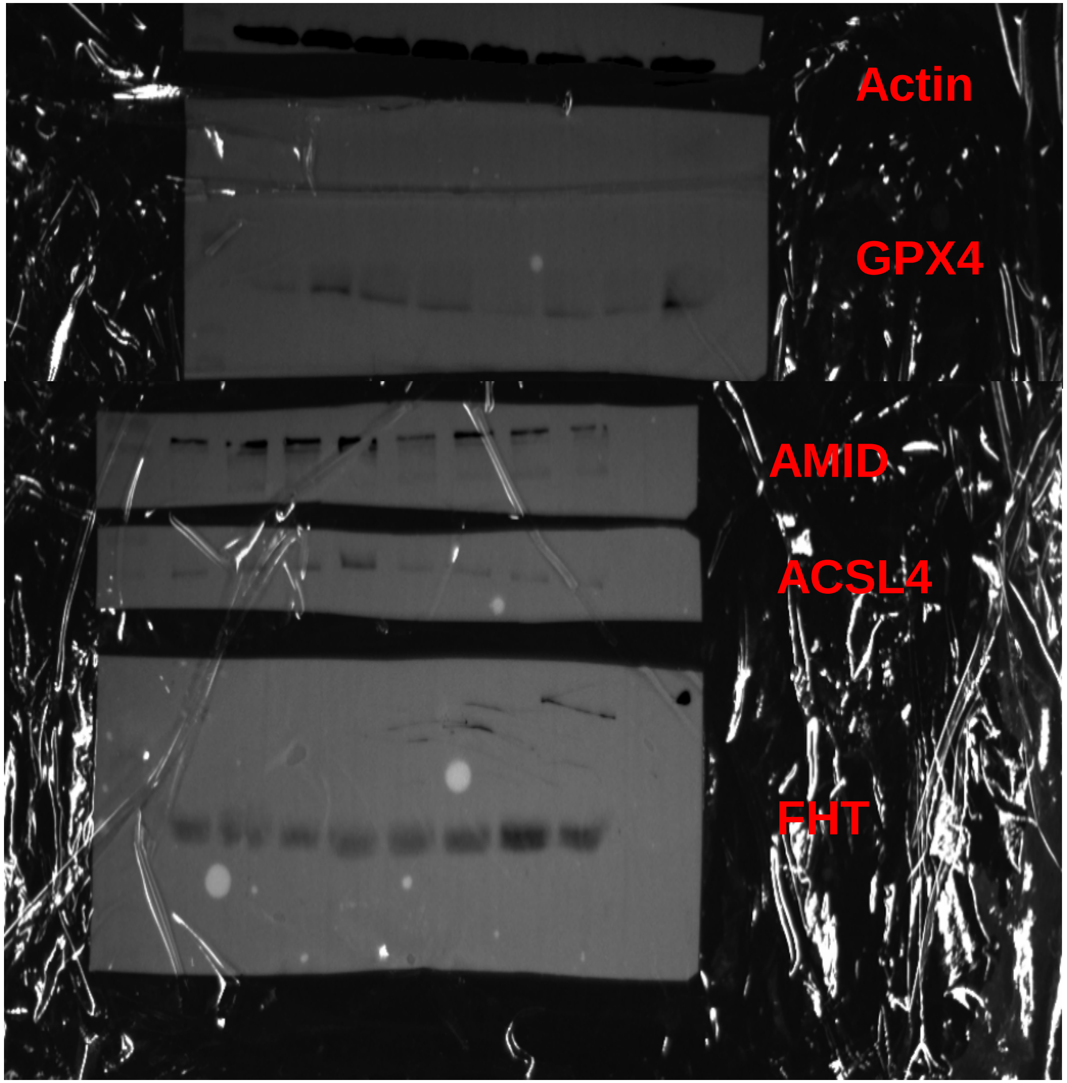
Actin
GPX4
AMID
ACSL4
FHT

Figure 5D

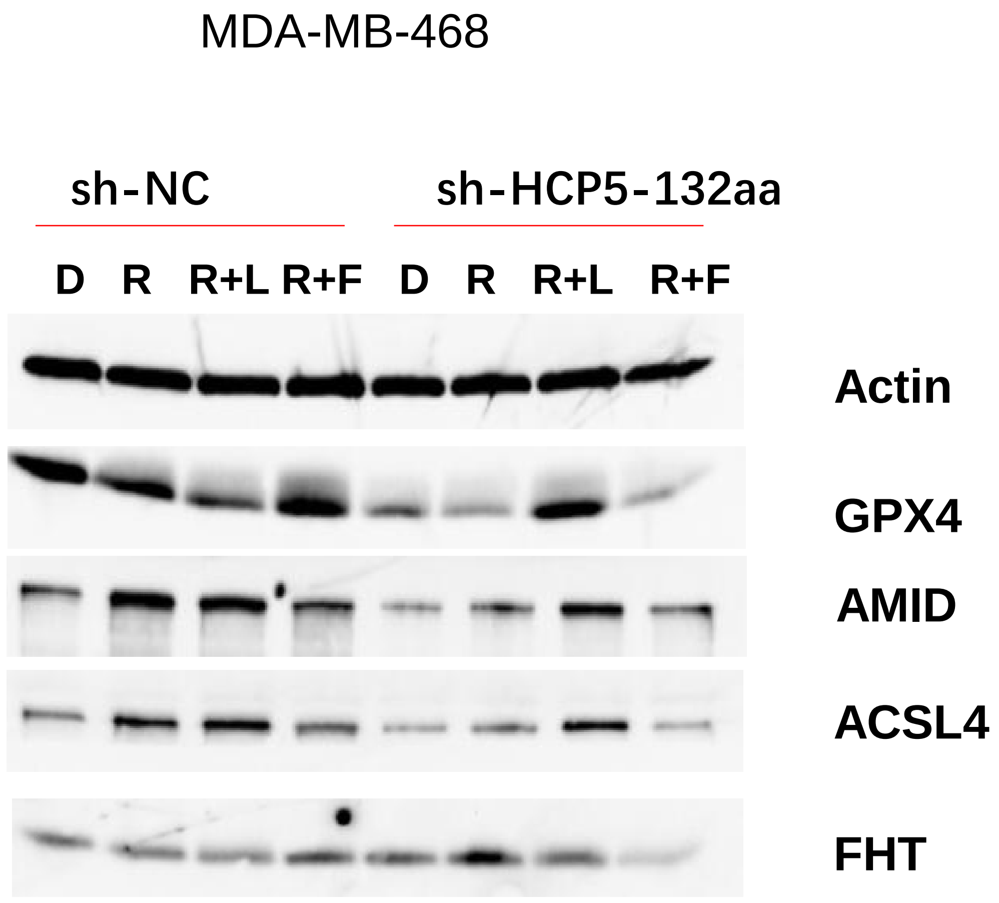
MDA-MB-468
sh-NC
sh-HCP5-132aa
D
R
R+L
R+F
D
R
R+L
R+F
Actin
GPX4
AMID
ACSL4
FHT

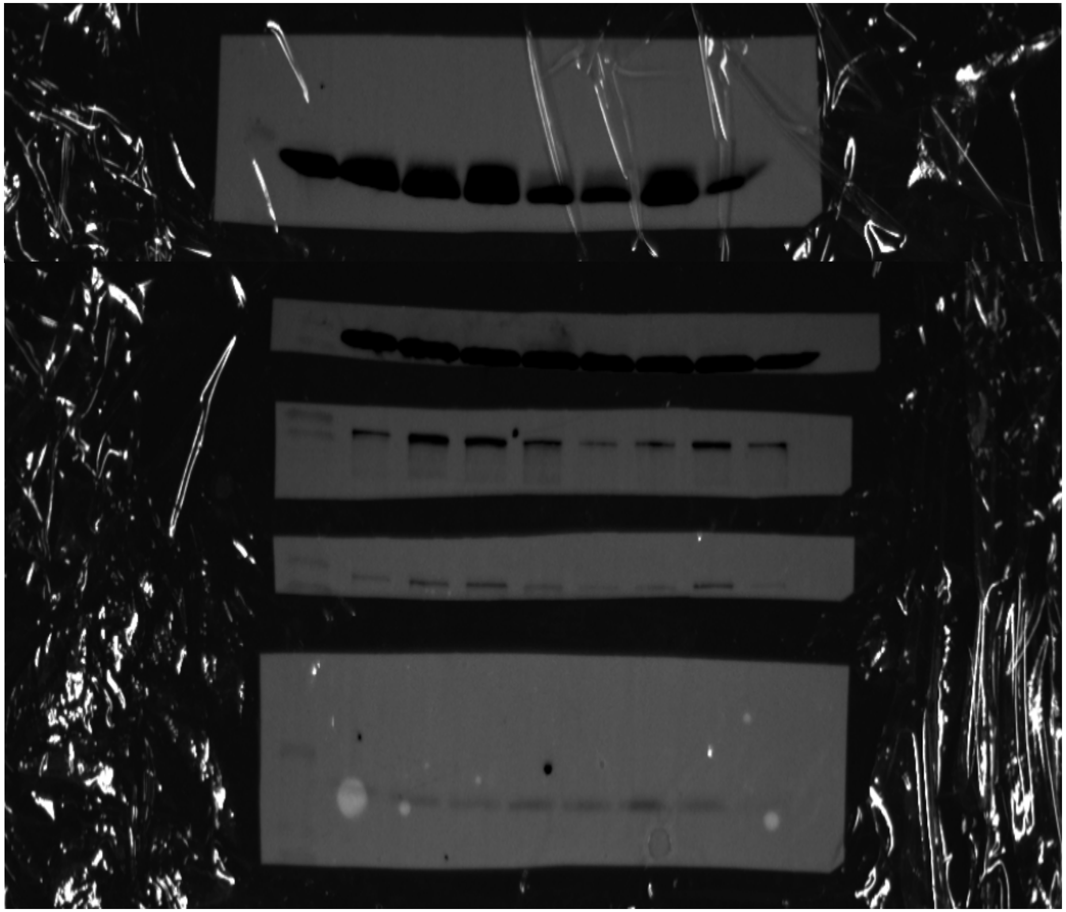

GPX4

Actin

AMID

ACSL4

FHT

Figure S3

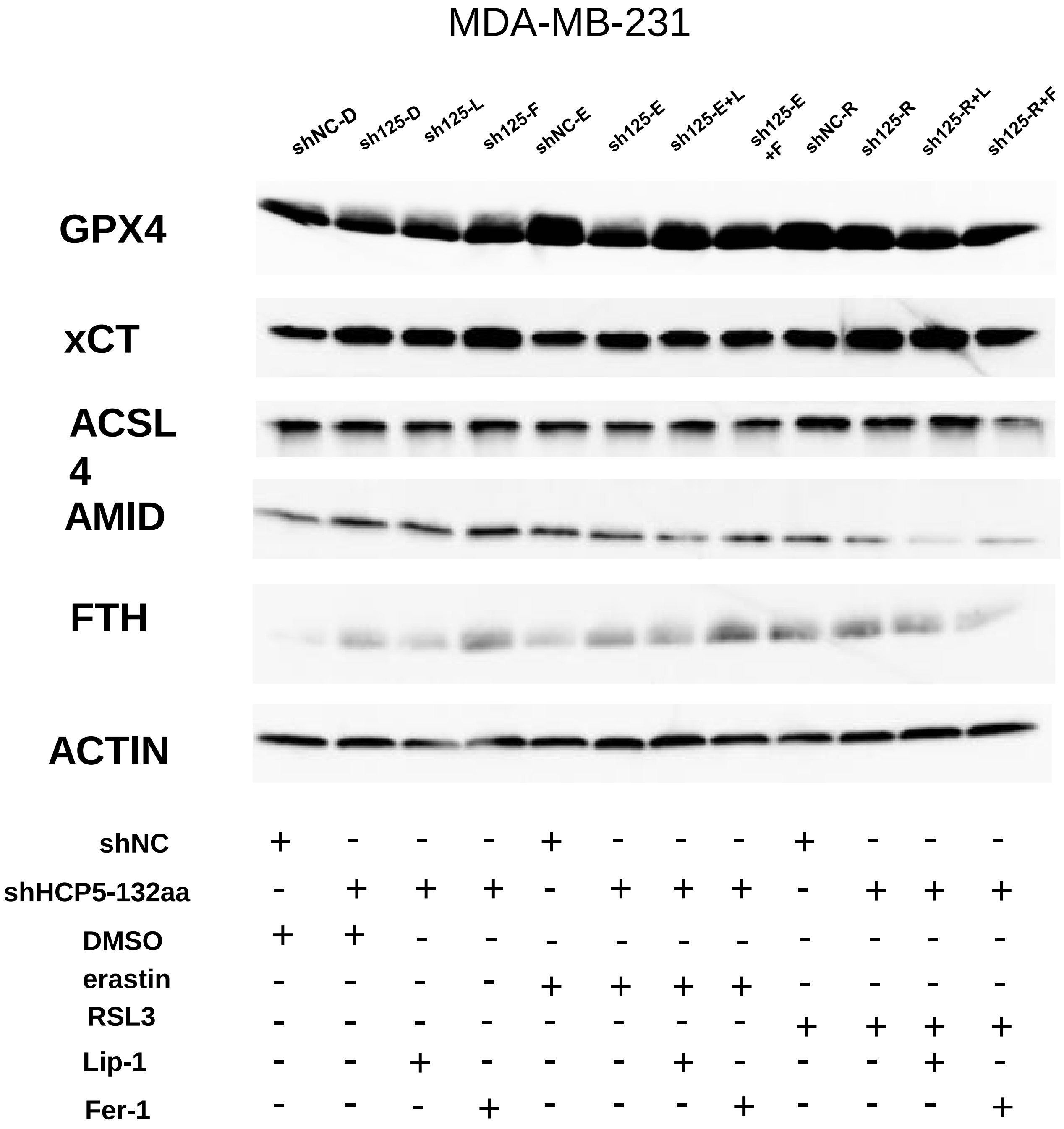
MDA-MB-231
shNC-D
sh125-D
sh125-L
sh125-F
shNC-E
sh125-E
sh125-E+L
sh125-E +F
shNC-R
sh125-R
sh125-R+L
sh125-R+F
GPX4
xCT
ACSL 4
AMID
FTH
ACTIN
shNC + - - - + - - - + - - -
shHCP5-132aa - + + + - + + + - + + +
DMSO + + - - - - - - - - - -
erastin - - - - + + + + - - - -
RSL3 - - - - - - - - + + + +
Lip-1 - - + - - - + - - - + -
Fer-1 - - - + - - - + - - - +

FTH

GPX4

AMID

ACSL4

xCT

Nrf2

Actin

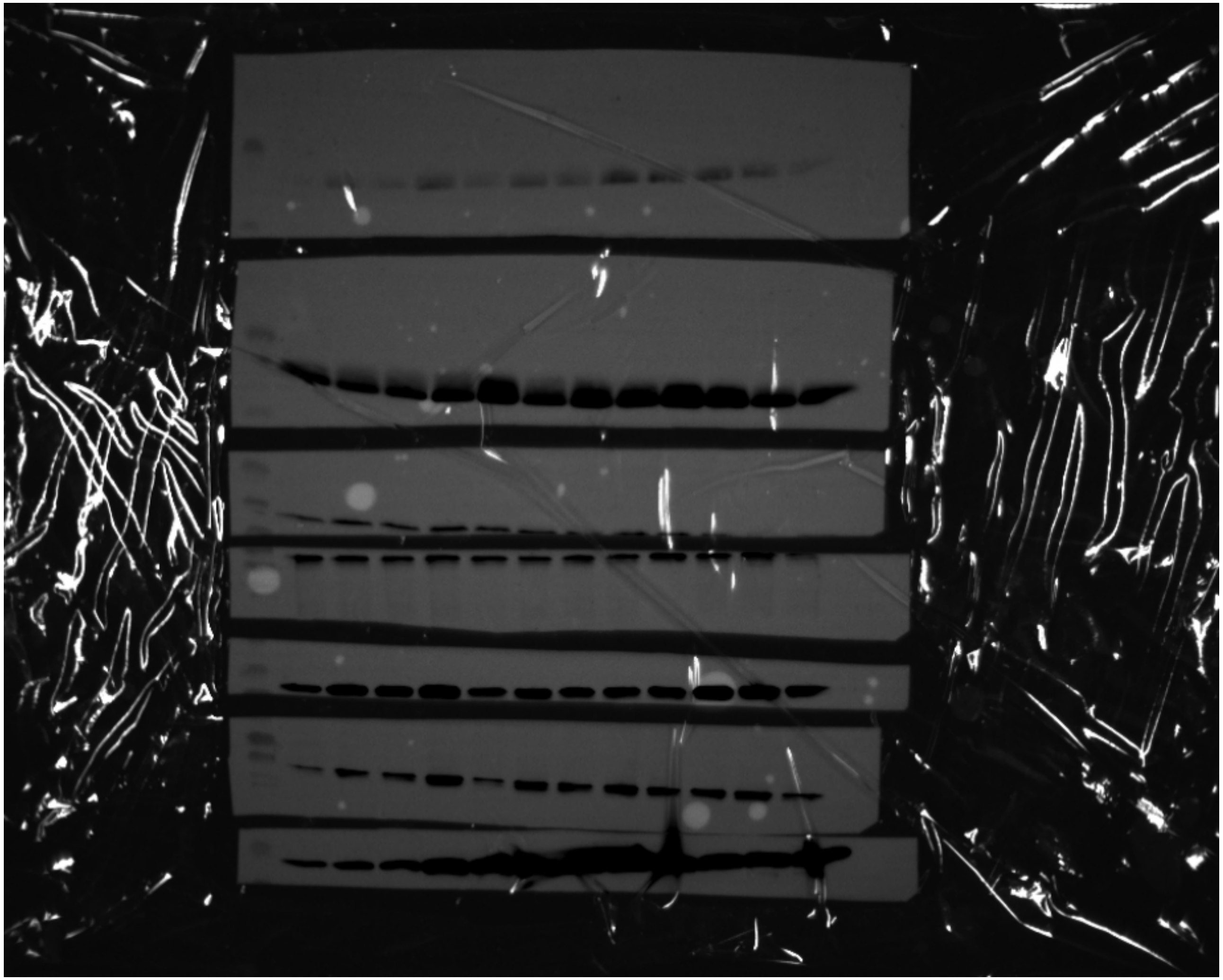

Figure S3

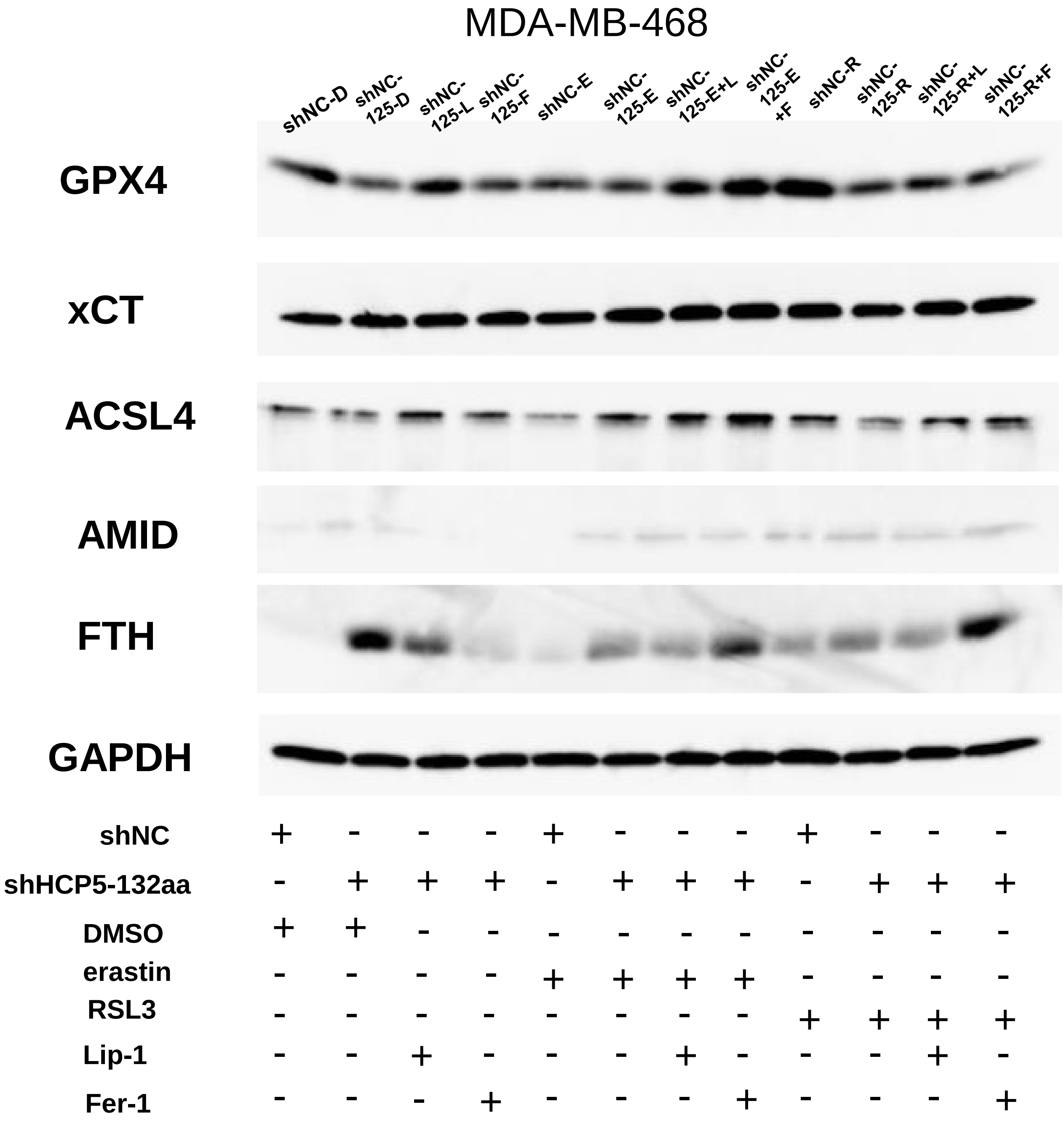
MDA-MB-468
shNC-D
shNC-125-D
shNC-125-L
shNC-125-F
shNC-E
shNC-125-E
shNC-125-E+L
shNC-125-E+F
shNC-R
shNC-125-R
shNC-125-R+L
shNC-125-R+F
GPX4
xCT
ACSL4
AMID
FTH
GAPDH
shNC + - - - + - - - + - - -
shHCP5-132aa - + + + - + + + - + + +
DMSO + + - - - - - - - - - -
erastin - - - - + + + + - - - -
RSL3 - - - - - - - - + + + +
Lip-1 - - + - - - + - - - + -
Fer-1 - - - + - - - + - - - +

468

FTH

GPX4

AMID

ACSL4

Nrf2

xCT

GAPDH

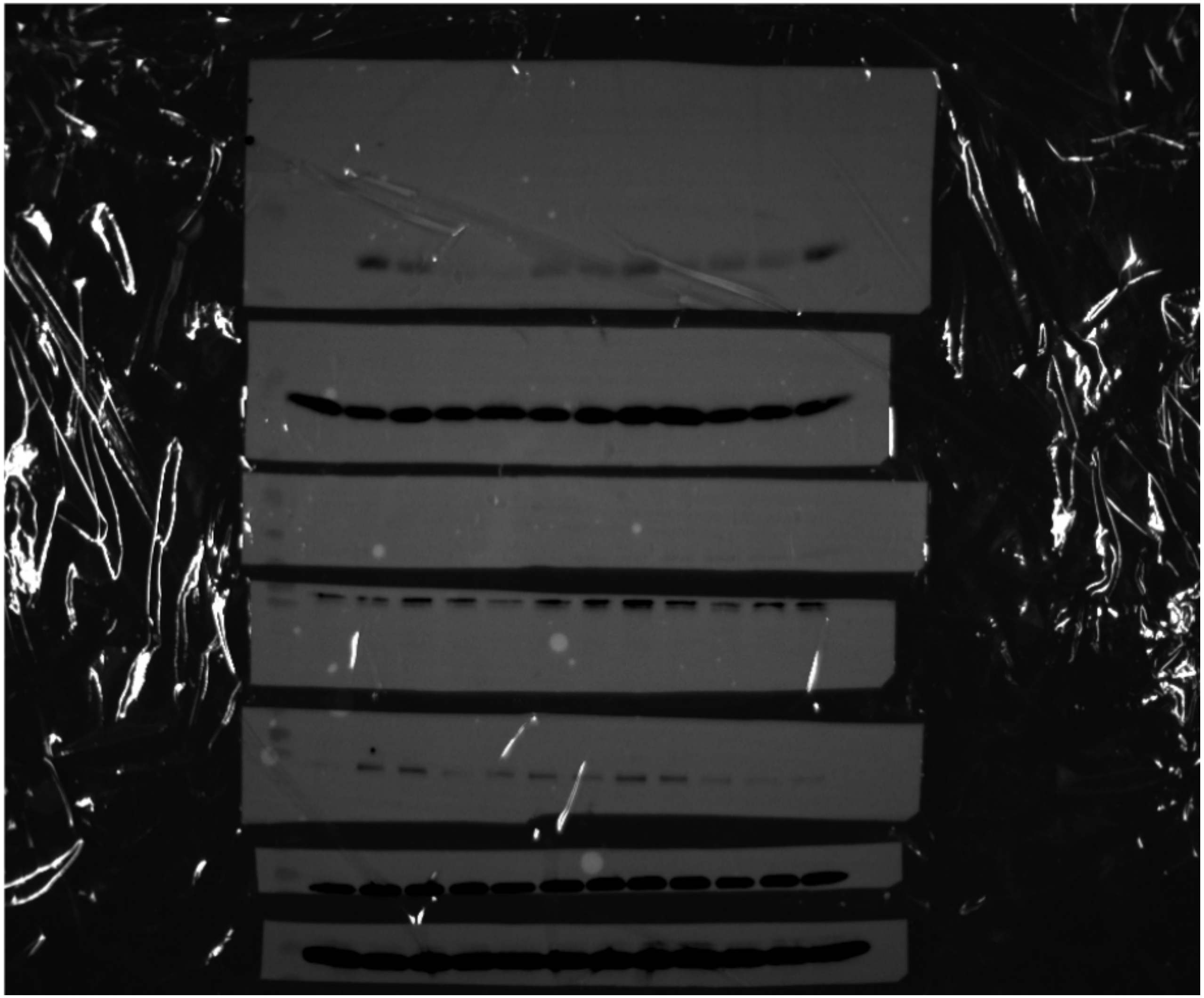

Supplement: Supplementary file 1 [file cancers-15-01880-s001.zip › cancers-2129240-supplementary-WB raw data.pdf]
